# Comparison of commercial 1Tx32Rx vs. 8Tx32Rx head coils for routine 7T neuroimaging

**DOI:** 10.3389/fnimg.2026.1736950

**Published:** 2026-03-25

**Authors:** Carina Graf, Belinda Ding, Catarina Rua, Krzysztof Klodowski, Christopher T. Rodgers

**Affiliations:** 1 Wolfson Brain Imaging Centre, Department of Clinical Neurosciences, University of Cambridge, Cambridge, United Kingdom; 2 Cambridge Centre for Parkinson-plus, University of Cambridge, Cambridge, United Kingdom; 3 Antaros Medical, Goco House, Mölndal, Sweden

**Keywords:** 7T MRI, neuroimaging, parallel transmit, transmit-receive head coil, ultra-high field MRI

## Abstract

**Introduction:**

Human 7T MRI systems are manufactured by three vendors (Siemens, Philips, and GE) who all provide equivalent head coils from the same 3rd party manufacturer. Furthermore, many 7T MRI sites have two head coils available for neuroimaging: a 1Tx32Rx head coil for conventional single-channel transmit imaging and an 8Tx32Rx head coil for parallel-transmit (pTx) imaging.

**Methods:**

We compared the performance of these coils in six healthy volunteers. All scans were done on a 7T MRI (MAGNETOM Terra, Siemens, Germany). We tested seven sequences in wide use at our centre: B_0_ and B_1_^+^ mapping, anatomical T_1_-weighted MP2RAGE, R_2_*-mapping, single-voxel spectroscopy (MRS), echo-planar imaging time series, and diffusion tensor imaging (DTI). Sequences were run unmodified and without any pTx pulses.

**Results:**

Data quality is comparable for both coils. The 8Tx32Rx coil had improved B_1_^+^ in inferior brain regions, enhanced spinal cord visibility in the cervical spine on anatomical MP2RAGE, higher SNR in MRS of the brainstem, and more defined fitted white matter tracts in the DTI images. All sequences showed acceptable data quality with the 8Tx32Rx coil.

**Conclusion:**

It is reasonable to substitute the 8Tx32Rx coil for the 1Tx32Rx coil for standard neuroimaging protocols. This will enable advanced parallel transmit sequences to be added to protocols with minimal disruption.

## Introduction

Ultra-high field (UHF) MRI at magnetic field strengths of 7T and above offers higher spatial resolution, improved contrast-to-noise ratio and can enable accelerated acquisitions for human imaging (Beisteiner et al., 2011; Ladd et al., 2018). At the time of writing, more than 125 human MRI scanners worldwide operate at or above 7T (Huber, n.d.) with magnets being supplied by all three leading vendors of UHF - Siemens Healthcare, Philips, General Electric, with two approved and released for clinical use (U.S. Food and Drug Administration, 2017; Medgadget, 2020).

Despite the growing popularity of UHF MRI systems, especially for neuroimaging, UHF systems currently do not incorporate integrated RF transmit coils, warranting the procurement of dedicated/local transmit and receive coils (Kraff and Quick, 2019). For neuroimaging at 7T, a widely used coil is a commercially available 1Tx32Rx head coil, which is resold by all three of the UHF vendors.

One challenge of UHF-MRI is that MR images are prone to signal dropouts due to B_1_ inhomogeneity. Parallel transmit (pTx) seeks to mitigate the challenges of B_1_^+^ transmit field homogeneity and RF heating (Padormo et al., 2016), with pTx capabilities being available for systems from all three leading vendors. As part of the pTx system, specialised coils are required which can also be procured through the vendors but are frequently manufactured by 3rd party companies. In our case, we are using a commercially available 8Tx32Rx coil manufactured by the same vendor as the 1Tx32Rx coil. This coil is also resold by all three vendors and is likely to be the most popular commercial coil for pTx neuroimaging at 7T.

Alongside the leading vendors, the UHF research community has also made considerable efforts toward bringing pTx into the clinical research setting, though a ‘gap’ remains between MR technical development and clinical research applications of pTx at 7T (Williams et al., 2023). This is because current published pTx studies predominantly focus on a single sequence and are conducted in a small sample of healthy volunteers (Wu et al., 2018; Wu et al., 2019). Although these sequences have shown great potential in improving image quality, larger-scale clinical research at UHF tends to involve a variety of sequences.

Unfortunately, pTx implementations are not yet available for all sequences in routine neuroimaging protocols. This has limited clinical adoption at our centre, as investigators were reluctant to transition established protocols to the 8Tx32Rx coil without evidence that core sequences would perform at least equivalently in the absence of pTx. Such reluctance is consistent with well-described risk-aversion and loss-aversion behaviours in clinical research, where perceived risks of disrupting established workflows outweigh potential gains from innovation, even when the objective risk is low (Eichler et al., 2013).

Fortunately, the pTx system combined with the 8Tx32Rx coil also offers the possibility to run sequences in the circularly polarised (CP) mode, where the amplitudes and phases of each transmit channel are set to mimic imaging protocols acquired with the single-transmit 1Tx32Rx coil. This is also known as the ‘TrueForm’ mode on the MAGNETOM Terra scanner. This capability offers the benefit that subjects can be scanned with the pTx system, and sequences without pTx capabilities can be run in the ‘TrueForm’ mode; at the same time, sequences with pTx capabilities can be added easily to the protocol without a coil change. We are using this strategy to encourage adoption of pTx neuroimaging methods in clinical research at Cambridge.

However, no published data have compared the image quality between the 1Tx32Rx head coil for conventional 1Tx32Rx imaging and an 8Tx32Rx head coil which is capable of pTx imaging. This has led to some apprehension about migrating clinical neuroimaging studies from the 1Tx32Rx system to the pTx capable 8Tx32Rx system.

As an aside, we would like to note for our MRI physicist readers that this apprehension is not unreasonable. Rather it represents the different perspective of translational clinical researchers and clinical trialists. For them, MRI is just one component of an overall trial design. It is often perceived as better to use a “tried and true” endpoint measure rather than one that may technically be better (e.g. more accurate, cheaper or more tolerable) because it adds risk that if it fails or if the clinical community feel it is not equivalent then the whole trial becomes moot. This risk aversion is well documented, especially in the context of the statistical methods for clinical trials where there is also a very strong risk aversion (Eichler et al., 2013).

Thus, we set out to compare the performance of seven common sequences for neuroimaging and spectroscopy in healthy volunteers scanned on both systems with the respective head coils. We aimed to understand the data quality implications of running routine neuroimaging protocols in CP mode on the 8Tx32Rx system rather than with the 1Tx32Rx system.

In our study, both coils used were supplied by the vendor and manufactured by a 3rd party. As previously stated, these coils are also resold by all three vendors and are likely to be the most popular head coils for 7T MRIs. Hence, we believe our findings will be of broad utility among 7T sites and offer a simple and practical route to introducing pTx sequences in clinical protocols.

## Methods

### MR methods

**Figure 1:**
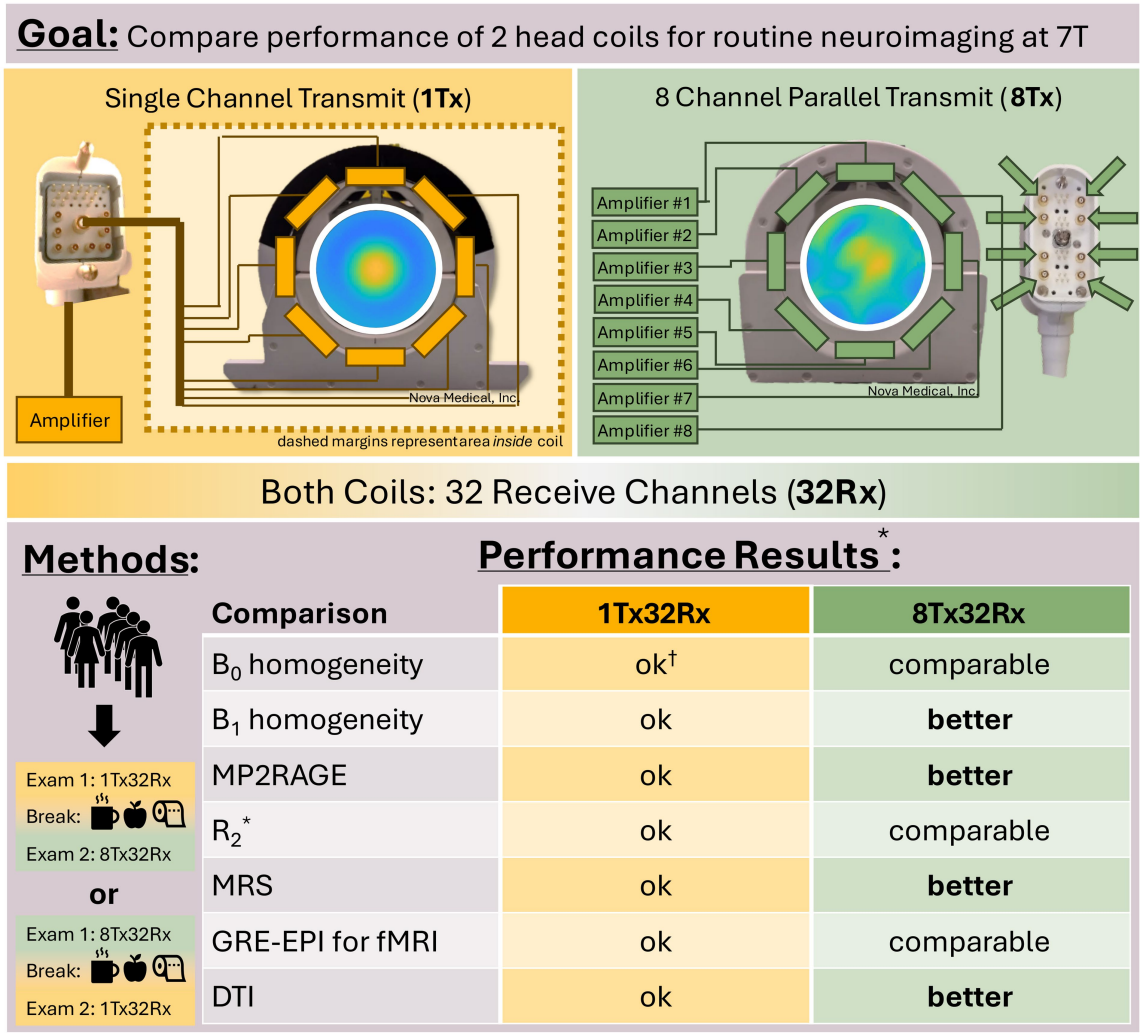
**Experimental set-up and results summary**. Study design and results overview. *This table refers to standard neuroimaging of the cerebrum. For studies imaging cerebellum, brainstem, or cerebral spine it is likely even more important to adopt the 8Tx32Rx head coil or use a custom-designed coil such as a spine coil. ^†^o*k* = providing satisfactory performance using the 1Tx32Rx system to warrant inclusion in routine clinical neuroimaging research protocols.

Six volunteers (2 female; age = 31.3 ± 4.5 years; BMI = 21.3 ± 1.2 kg/m^2^) were scanned twice on a MAGNETOM Terra 7T MRI scanner (Siemens Healthcare, Germany, VE12U software) using the vendor-supplied 1Tx32Rx and 8Tx32Rx head coils (both from Nova Medical Inc., USA, Figure 1). Scans with the 1Tx32Rx head coil were performed using the single transmit host computer, and scans with the 8Tx32Rx head coil were performed using the parallel transmit host computer. All participants were positioned supine on the scanner bed with earplugs and immobilisation cushions to minimise motion. Head positioning was standardised by gently positioning the head to touch the top of the RF coil to maximise coverage consistency across subjects. Laser alignment was performed using the centre mark on the respective RF coils to ensure consistent positioning relative to isocentre. The B_1_ shim mode was set to ‘TrueForm’ on the pTx host computer, implying that the 8Tx32Rx coil was running in the CP mode. This mode sets equal amplitudes for every channel and equally spaced phases to emulate the birdcage transmission of the 1Tx32Rx head coil. Both protocols were acquired in 1st Level mode. Pulse sequences and protocols were run without modification on the two host computers.

Scans were performed on the same day with a scheduled 15-min break to allow switching between the systems. The time taken for the host switch was recorded and also includes the time required for the coil change on the scanner bed. The order of scanning between the two coils was randomised. A summary of the sequences ran in each subject can be found in the Supplementary Table S1.

### Data acquisition

For all scans, the transmit voltage was determined automatically by the scanner. B_0_-shimming was performed using the scanner’s auto-calibration routine followed by manual tuning of shim currents to minimise measured linewidths following procedures developed in the UK7T Network Travelling Head Study (Clarke et al., 2020). All imaging scans had the same shim volume.

B_0_ maps were acquired using a 3D dual-echo gradient-recalled echo (GRE) sequence with an in-plane image resolution of 2.0 × 2.0 mm^2^ and a slice thickness of 1.6 mm. Other parameters include: 1.11/3.06 ms echo times (TE), 5.5 ms repetition time (TR), 10° non-selective excitation, and generalised autocalibrating partial parallel acquisition (GRAPPA) acceleration factor of 2, 54 s acquisition time (TA). Both echoes had a readout bandwidth of 910 Hz/Px with monopolar readout gradients.

Flip angle mapping was performed using a saturation-prepared turbo-FLASH imaging sequence with 2.0 mm isotropic resolution (TE = 2.28 ms, TR = 18,750 ms, 90° sinc preparation pulse, 8° readout flip angle, 490 Hz/Px readout bandwidth, TA = 56 s). Initially, we acquired 35 slices with a distance factor of 100%. After three volunteers, we increased to 50 slices for better brainstem coverage (see Supplementary Table S1).

T_1_-weighted structural images were acquired using a dual magnetization-prepared rapid gradient echo (MP2RAGE) at 0.75 mm isotropic resolution, in sagittal orientation over a 240 × 225 × 252 mm^3^ (read, phase and slice axes) field of view, and GRAPPA acceleration factor of 3 in the phase encoding direction. Other parameters were: 5°/6° flip-angles, TR = 4,300 ms, TE = 1.94 ms, inversion times = 840/2,370 ms, and 250 Hz/Px readout bandwidth, TA = 8 min 50s.

R_2_* maps were acquired with a 3D multi-echo gradient-echo sequence at 0.7 mm isotropic resolution over 224 slices with four echoes. Imaging parameters include: TE = 4.58 ms for the first echo, TR = 27 ms, 3.32 ms echo spacing, 15° flip angle, 430 Hz/Px bandwidth, and 2 × 2 GRAPPA acceleration, TA = 6 min 55 s.

The single-voxel MR spectroscopy acquisition followed the CMRR MRS C2P recommended protocol using FASTESTMAP. B_1_^+^ and water suppression voltage were adjusted with a two-step iterative parameter sweep in short, non-water suppressed acquisitions (total time for adjustment scans: 6 min 15 s). The 12x12x20mm^3^ voxel was placed at the ponto-medullary junction of the brainstem. The main acquisition consisted of 120 water-suppressed spectra using a semi-LASER sequence with GOIA-WURST pulses, 26 ms TE and 5,000 ms TR, 10 min 45 s TA. Four non-water suppressed spectra were acquired for water scaling and eddy-current correction, respectively.

Simultaneous multi-slice (SMS) GRE-EPI (gradient-recalled echo planar imaging) data were acquired in five subjects with a sequence previously published by Moeller et al. (2010) and Jia et al. (2021). A total of 100 volumes were acquired in the transverse orientation at 1.5 mm isotropic resolution, with 96 slices covering the whole brain. Imaging parameters include: TE = 21 ms, TR = 1,689 ms, a 55° excitation flip angle, TA = 3 min 16 s and A > P phase encoding. Accelerated imaging was performed with an acceleration factor of 2 in the phase encoding direction and a multi-band factor of 3. An additional five-volume run was performed with a reverse phase-encoding direction for distortion correction (TA = 35 s).

Diffusion tensor imaging (DTI) was acquired in three subjects using single-shot spin-echo EPI with 2.0 mm isotropic resolution and 83 slices covering the whole brain. The data were collected from six directions with two different diffusion gradients (b = 500, 1,000 s/mm^2^) and one without diffusion weighting (b = 0 s/mm^2^). Other imaging parameters include: TE = 41 ms; TR = 7,000 ms; phase partial Fourier = 6/8, GRAPPA acceleration factor of 3, TA = 5 min 10s. Three averages were collected with each *b*-value and diffusion direction. An additional non-diffusion weighted image was collected with the reverse phase-encoding direction for distortion correction (TA = 58 s).

Further sequence details are listed in the Supplementary Data Sheets 1, 2.

### Data analysis

DICOM images exported from the scanner were converted into NIFTI format (dcm2nii, MRIcron, 2014 version (Li et al., 2016)). Unless otherwise specified, subsequent data analysis was conducted using the FMRIB Software Library (Jenkinson et al., 2012) (FSL, v6.0.3, FMRIB, UK) on images and quantitative maps in the native subject space.

#### Structural image processing

Three magnitude images were obtained from the MP2RAGE acquisition: the first inversion image (INV1), the second inversion image (INV2), and the combined T_1_-weighted structural image (UNI). Cortical reconstruction and volumetric segmentation of the UNI MP2RAGE images were performed using FreeSurfer (v7.1.0).

#### B_0_ and B_1_^+^fieldmaps processing

B_0_ maps were reconstructed from the magnitude and phase images with ‘fsl_prepare_fieldmap’. Voxel-wise “normalised B_1_^+^ maps” were calculated as the ratio of the measured flip angle divided by the expected flip angle, as previously described by Clarke et al. (2020).


$$\text{normalised}\;B_{1}^{+}=\frac{\text{measured flip angle}}{\text{expected flip angle}}$$ (1)

Voxel-wise δB_1_^+^ for each participant was calculated using:


$$\delta B_{1}^{+}(8\mathrm{Tx}-1\mathrm{Tx})=\frac{B_{1}^{+}(8\mathrm{Tx})-B_{1}^{+}(1\mathrm{Tx})\;}{B_{1}^{+}(8\mathrm{Tx})+B_{1}^{+}(1\mathrm{Tx})}\times 200\%$$ (2)

#### Quantitative neuroimaging sequences

R_2_^*^ fitting was performed on the multi-echo T_2_^*^ weighted magnitude data for each subject and session, using the Auto-Regression on Linear Operations (ARLO) method implemented in MATLAB (Pei et al., 2015). The voxel-wise δR_2_^*^ maps were calculated similarly to Equation 2, as the percentage difference between the two R_2_^*^ values in each voxel.

Individually saved shots for MRS were phase-corrected, eddy-current corrected and frequency aligned in MRSpa (version 1.5 g) before fitting in LCModel (v6.3-1R) (Provencher, 1993) with a sequence-specific basis set including 26 simulated metabolites and a measured macromolecular baseline (Terpstra et al., 2016). Water scaling in LCModel using the non-water suppressed acquisitions was used for concentration referencing with concentrations corrected for the volumetric fraction of cerebrospinal fluid (f_CSF_) as determined by segmentation of the UNI-MP2RAGE with FSL-MRS and FSL’s FAST (Clarke et al. 2021). Metabolite concentrations are reported in arbitrary units (a.u.). LCModel determined linewidth and SNR were used as overall spectral quality criteria, while Cramer-Rao Lower Bounds (CRLBs) were used as a marker for fit accuracy of individual metabolites. Full details on the acquisition, processing and fitting of MRS data according to the Minimum Reporting Standard in MRS (MRSinMRS) (Lin et al., 2021) can be found in Supplementary Table 2.

GRE-EPI data were corrected for motion (FSL’s ‘mcflirt’ (Jenkinson et al., 2002)). Distortion correction was performed with the reversed gradient approach using a five A-P volume mean and the five reverse encoded P-A volumes using FSL’s ‘topup’ (Andersson et al., 2003) to obtain distortion fields used to correct all corresponding functional magnetic resonance imaging (fMRI) runs. A boundary-based registration method (FSL’s ‘epi_reg’) was used to register each distortion-corrected EPI run to the MP2RAGE (Greve and Fischl, 2009). Voxel-wise temporal signal-to-noise ratio (tSNR) was computed as the ratio of the temporal mean to the temporal standard deviation in the series.

Diffusion imaging series were corrected for distortion (‘topup’ in FSL, see above). Distortion corrected and brain extracted (see below) images were then corrected for eddy current effects (FSL’s ‘eddy’ (Andersson and Sotiropoulos, 2016)), followed by N4 bias field correction from the ANTs package (Tustison et al., 2010). For quantitative analysis, diffusion tensors (FSL’s ‘dtifit’) and first fibre orientation dispersion (FFD) was calculated using FSL’s ‘bedpostX’ from the corrected images (Jbabdi et al., 2012).

#### Registration pipeline and region of interest (ROI) analysis

In the main instance UNI-MP2RAGE images were registered to the standard MNI152 brain using a linear 12 degree-of-freedom affine registration (FSL’s FLIRT (Jenkinson et al., 2002)). Linear registration was performed with a correlation ratio cost function, search limits of ±90° for all three rotation axes and tri-linear interpolation. Each subject registration was manually verified.

Other, low-resolution images for quantitative maps (B_0_, B_1_^+^, R_2_^*^, GRE-EPI, DTI) were first linearly registered (FLIRT) to their respective MP2RAGE-INV2 before concatenating these transformations with the transformation matrix to map to standard space. Except for R_2_^*^, where a rigid affine registration method was used to register the individual T_2_^*^ data to the UNI-MP2RAGE images (ANTs (v2.1.0) registration; cross-correlation similarity metric).

Brain masking in native image space proved difficult due to signal dropout across sequences. Thus, we generated subject brain masks by linearly mapping the MNI standard brain mask to individual lower resolution image spaces, via the MP2RAGE-INV2. This prevented differences in masking due to signal dropout. Histograms of B_0_ and normalised B_1_^+^ across the whole brain were plotted as defined by these brain masks.

ROI analysis was performed in nine anatomical ROIs defined in the Harvard-Oxford structural atlas (Goldstein et al., 2007; Desikan et al., 2006; Frazier et al., 2005; Makris et al., 2006). The ROI binary masks were mapped onto the native imaging spaces by applying the final aggregate transformation matrix to standard space in reverse. The following metrics were compared in each ROI for each subject: mean and standard deviation of normalised B_1_^+^, R_2_^*^ and GRE-EPI tSNR.

### Statistical analysis

Paired two-tailed Student’s t-tests were used for all statistical testing of MR data, except first fibre orientation dispersion distributions which displayed moderate right skewness (above 0.75) and substantial deviation from normal distribution (kurtosis above 2.75), in which case the Mann–Whitney *U*-test was used. Outliers were removed using the quartile method. The Benjamini–Hochberg procedure was used to control for false discovery rates in the case of multiple comparisons within each MR modality. Preliminary results from this cohort have previously been presented at ISMRM 2022 (Graf et al., 2022; Ding et al., 2022).

## Results

On average, switching between the 1Tx32Rx to 8Tx32Rx system took 6 min 16 s. Visual inspection of the unprocessed images and spectra did not reveal untypical or unexpected artefacts, and as such no data were excluded from further analysis.

**Figure 2:**
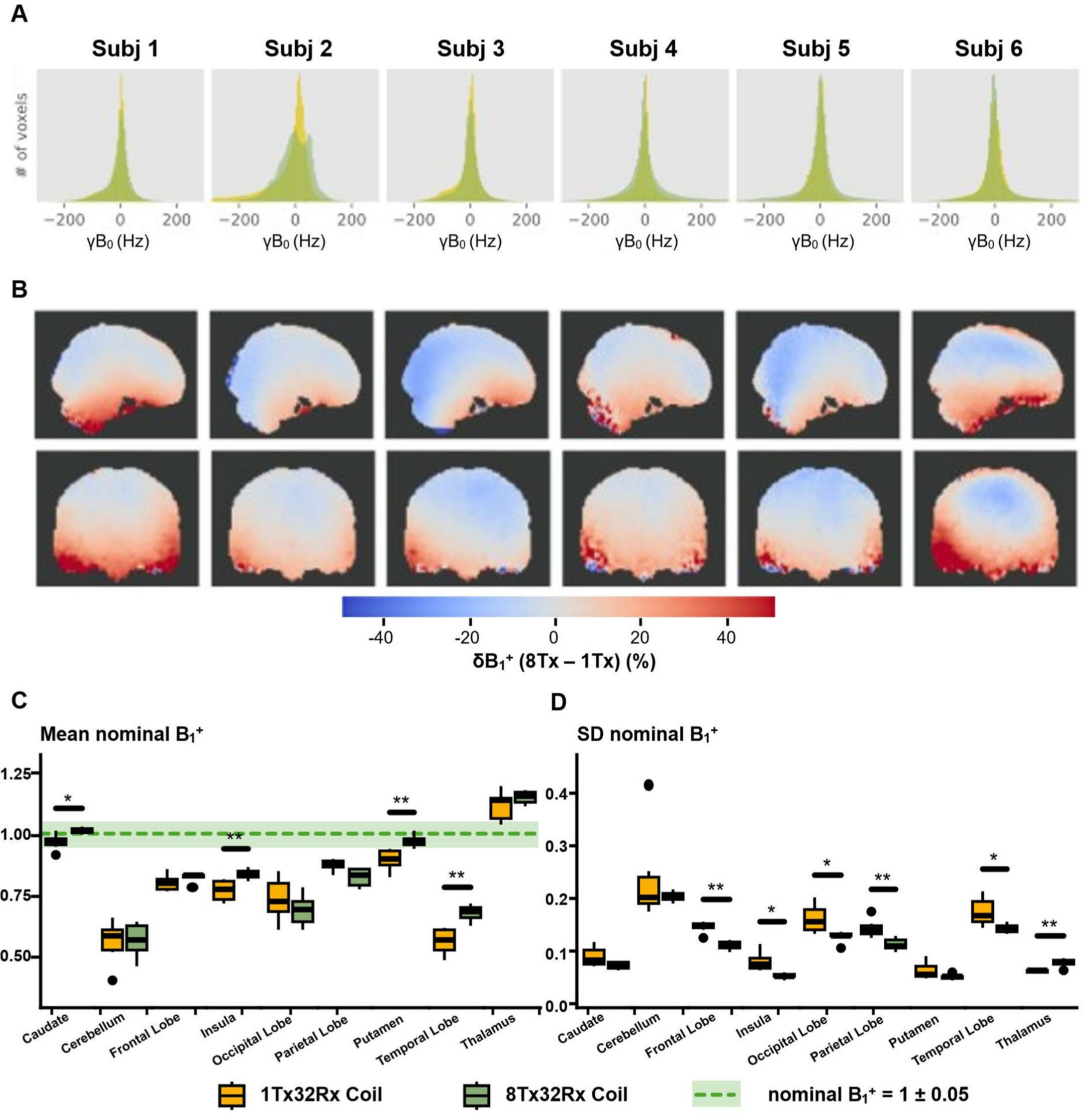
**B_0_ and B_1_^+^**. **(A)** Histograms of B_0_ homogeneity (γΔB_0_) indicate similar homogeneity between the 1Tx32Rx (yellow) and 8Tx32Rx (green) coil. **(B)** Sagittal (top) and coronal (bottom) views of δB_1_^+^(8Tx – 1Tx) maps for all six volunteers. The maps were normalised into MNI space. **(C)** Mean normalised B_1_^+^ in the nine anatomical ROIs defined by the MNI atlas. Four ROIs (caudate, insula, putamen, temporal lobe) had higher mean nominal B_1_^+^ (*p* < 0.05) with nominal B_1_^+^ closer to 1 (green highlight). **(D)** Six ROIs had lower standard deviations for nominal B_1_^+^ in the 8Tx32Rx coil. Standard deviation values across the ROI are used as a surrogate to indicate B_1_^+^ homogeneity. * *p* < 0.05, ** *p* < 0.01, between 1Tx32Rx and 8Tx32Rx results, after adjusting for multiple comparisons.

Figure 2A shows histograms of whole-brain γΔB_0_. Across all volunteers, the mean whole-brain averaged γΔB_0_ was −6.3 ± 12.60 Hz for the 1Tx32Rx coil and 13.9 ± 26.61 Hz for the 8Tx32Rx coil (*p* = 0.076); while the mean standard deviation of γΔB_0_ was 86.86 ± 13.51 Hz for the 1Tx32Rx coil and 125.46 ± 49.76 Hz for the 8Tx32Rx coil (*p* = 0.132). B_0_ maps for all participants can be found in Supplementary Figure S1.

While occipital and parietal lobes had mean normalised B_1_^+^ (Equation 1) closer to 1 when using the 1Tx32Rx coil, the differences were not statistically significant (*p* > 0.12, Table 1, Figures 2B,C and Supplementary Figure S2). In contrast, we saw significantly higher normalised B_1_^+^ values in the caudate, insula, putamen, and temporal lobes with the 8Tx32Rx coil (Figures 2B,C) (*p* < 0.01, Table 1). To ensure that the improvement in mean normalised B_1_^+^ values did not come with a penalty on the homogeneity, we also assessed the standard deviation of normalised B_1_^+^ in each ROI (i.e., a measure of B_1_^+^ homogeneity). The 8Tx32Rx coil showed significantly lower standard deviations of normalised B_1_^+^ in six out of nine ROIs, indicating more homogeneous B_1_^+^ (Figure 2D, Table 1).

**Table 1:** Summary of all MRI modalities.

Modality	Region of interest/MRS metric	Within subject mean			Within subject variation		
		1Tx32Rx	8Tx32Rx	*p* (corrected)	1Tx32Rx	8Tx32Rx	*p* (corrected)
B_0_ mapping^a^ (Hz)	Whole brain	−6.3 ± 12.60	13.9 ± 26.61	0.076	86.86 ± 13.51	125.46 ± 49.76	0.132
B_1_^+^ (normalised)	Caudate	0.98 ± 0.03	1.02 ± 0.01	0.01 (*)	0.092 ± 0.019	0.076 ± 0.006	0.06
	Cerebellum	0.57 ± 0.09	0.57 ± 0.07	0.92	0.240 ± 0.090	0.206 ± 0.009	0.37
	Frontal lobe	0.81 ± 0.03	0.83 ± 0.02	0.23	0.148 ± 0.012	0.114 ± 0.008	0.01 (*)
	Insula	0.78 ± 0.04	0.84 ± 0.02	<0.01 (*)	0.084 ± 0.019	0.056 ± 0.004	0.03 (*)
	Occipital lobe	0.74 ± 0.09	0.70 ± 0.06	0.21	0.164 ± 0.027	0.130 ± 0.011	0.03 (*)
	Parietal lobe	0.88 ± 0.02	0.83 ± 0.04	0.12	0.147 ± 0.017	0.115 ± 0.013	0.01 (*)
	Putamen	0.90 ± 0.05	0.98 ± 0.03	<0.01 (*)	0.067 ± 0.016	0.055 ± 0.004	0.1
	Temporal lobe	0.57 ± 0.06	0.68 ± 0.03	<0.01 (*)	0.177 ± 0.028	0.145 ± 0.008	0.03 (*)
	Thalamus	1.12 ± 0.07	1.16 ± 0.03	0.22	0.066 ± 0.003	0.081 ± 0.008	0.01 (*)
Brain volume (cm^3^)	Whole brain	1189.4 ± 83.6	1175.5 ± 81.6	0.28	–	–	
	Total GM	649.0 ± 43.8	643.5 ± 38.4	0.80	–	–	
	Total WM	521.7 ± 49.3	513.2 ± 48.9	0.24	–	–	
	Left cerebral WM	243.7 ± 22.5	241.5 ± 23.2	0.16	–	–	
	Right cerebral WM	245.0 ± 23.7	242.2 ± 25.1	0.24	–	–	
	Left cerebellum WM	16.1 ± 2.5	15.1 ± 0.8	0.71	–	–	
	Right cerebellum WM	16.8 ± 5.2	14.5 ± 1.0	0.76	–	–	
	Left lateral ventricle	9.0 ± 2.7	9.0 ± 2.9	0.86	–	–	
	Right lateral ventricle	7.6 ± 2.1	7.5 ± 2.3	0.71	–	–	
R_2_^*^ (1/ms)	Caudate	0.034 ± 0.002	0.034 ± 0.002	0.51	0.018 ± 0.001	0.018 ± 0.002	0.81
	Cerebellum	0.035 ± 0.001	0.035 ± 0.001	0.63	0.021 ± 0.002	0.022 ± 0.002	0.69
	Frontal lobe	0.034 ± 0.002	0.034 ± 0.002	0.51	0.025 ± 0.002	0.025 ± 0.002	0.69
	Insula	0.029 ± 0.002	0.028 ± 0.002	0.73	0.014 ± 0.001	0.014 ± 0.001	0.69
	Occipital lobe	0.040 ± 0.002	0.039 ± 0.002	0.10	0.023 ± 0.004	0.021 ± 0.004	0.19
	Parietal lobe	0.035 ± 0.002	0.035 ± 0.002	0.59	0.023 ± 0.002	0.022 ± 0.002	0.31
	Putamen	0.040 ± 0.003	0.039 ± 0.004	0.51	0.010 ± 0.001	0.010 ± 0.001	0.81
	Temporal lobe	0.038 ± 0.002	0.038 ± 0.001	0.10	0.029 ± 0.003	0.030 ± 0.003	0.11
	Thalamus	0.038 ± 0.003	0.037 ± 0.003	0.73	0.015 ± 0.001	0.015 ± 0.002	0.84
MRS	LW (Hz)	11.5 ± 2.0	11.5 ± 2.3	1.0	–	–	–	
	SNR	23.5 ± 3.7	29.3 ± 4.5	< 0.01 (*)	–	–	–	
	f_CSF_ (%)	2.1 ± 1.3	1.7 ± 1.0	1.0	–	–	–	
	Asc (a.u.)	54.70 ± 19.12	55.14 ± 25.84	1.00	9.16 ± 0.92	8.47 ± 0.81	1.00	
	Asp (a.u.)	68.98 ± 27.59	71.50 ± 11.50	1.00	15.65 ± 1.87	16.30 ± 1.96	1.00	
	Cr (a.u.)	95.64 ± 5.76	81.68 ± 17.00	1.00	8.78 ± 1.73	9.27 ± 1.34	1.00	
	GABA (a.u.)	45.27 ± 20.92	64.81 ± 9.12	1.00	11.07 ± 0.96	10.26 ± 0.91	1.00	
	GPC (a.u.)	87.82 ± 16.60	92.86 ± 15.58	1.00	3.21 ± 1.31	2.66 ± 1.20	1.00	
	GSH (a.u.)	34.95 ± 7.17	34.99 ± 13.65	1.00	4.46 ± 0.49	4.00 ± 0.29	0.83	
	GlcTau (a.u.)	74.54 ± 20.16	57.81 ± 11.82	0.56	8.74 ± 2.41	7.67 ± 1.54	1.00	
	Gln (a.u.)	53.55 ± 13.25	48.49 ± 21.91	1.00	9.54 ± 1.20	8.46 ± 0.56	0.73	
	Glu (a.u.)	173.01 ± 14.06	172.14 ± 11.90	1.00	8.90 ± 1.13	8.30 ± 0.62	1.00	
	Glx (a.u.)	226.56 ± 24.64	220.56 ± 16.42	1.00	13.54 ± 1.61	12.00 ± 1.57	1.00	
	Ins (a.u.)	320.40 ± 26.44	317.17 ± 39.35	1.00	6.87 ± 0.80	6.75 ± 0.40	1.00	
	NAA (a.u.)	281.55 ± 14.58	283.93 ± 7.16	1.00	6.06 ± 0.82	6.61 ± 1.37	1.00	
	NAAG (a.u.)	79.10 ± 4.66	78.78 ± 6.07	1.00	5.53 ± 0.68	5.20 ± 0.64	1.00	
	PCr (a.u.)	136.29 ± 14.67	142.21 ± 20.78	1.00	9.84 ± 1.71	9.68 ± 1.51	1.00	
	PE (a.u.)	75.43 ± 16.04	73.14 ± 24.25	1.00	12.91 ± 1.24	10.92 ± 0.74	0.16	
	Tau (a.u.)	56.74 ± 21.95	49.05 ± 12.75	1.00	7.58 ± 0.94	6.86 ± 0.34	1.00	
	sIns (a.u.)	11.47 ± 4.05	8.98 ± 7.22	1.00	1.99 ± 0.30	1.89 ± 0.35	1.00	
	tCho (a.u.)	96.29 ± 13.08	94.92 ± 14.13	1.00	2.07 ± 0.36	1.90 ± 0.28	1.00	
	tCr (a.u.)	231.93 ± 15.36	223.95 ± 5.00	1.00	4.64 ± 0.31	4.48 ± 0.10	1.00	
	tNAA (a.u.)	360.59 ± 18.36	362.28 ± 11.80	1.00	6.61 ± 1.52	6.63 ± 2.69	1.00
EPI tSNR	Caudate	26.9 ± 2.6	27.9 ± 2.5	0.53	10.5 ± 1.9	11.7 ± 2.1	0.03 (*)
	Cerebellum	24.2 ± 4.5	23.8 ± 1.8	0.84	13.3 ± 2.5	12.2 ± 1.3	0.2
	Frontal lobe	27.2 ± 3.7	27.5 ± 3.7	0.84	17.3 ± 2.0	18.5 ± 1.8	0.19
	Insula	31.7 ± 2.4	33.1 ± 2.3	0.53	8.9 ± 0.8	9.7 ± 0.6	0.04 (*)
	Occipital lobe	35.8 ± 5.6	33.5 ± 2.9	0.53	18.0 ± 1.6	16.2 ± 1.0	0.04 (*)
	Parietal lobe	34.2 ± 3.9	32.7 ± 2.2	0.53	19.0 ± 2.2	17.9 ± 1.5	0.17
	Putamen	25.8 ± 1.5	26.7 ± 2.6	0.53	6.3 ± 0.8	6.9 ± 0.8	0.01 (*)
	Temporal Lobe	22.6 ± 2.9	23.6 ± 2.7	0.53	13.6 ± 1.5	14.0 ± 1.3	0.53
	Thalamus	23.3 ± 1.5	23.8 ± 1.4	0.53	7.0 ± 0.4	7.6 ± 0.6	0.03 (*)

a reported as off-resonance γB_0_ (Hz).

**Figure 3:**
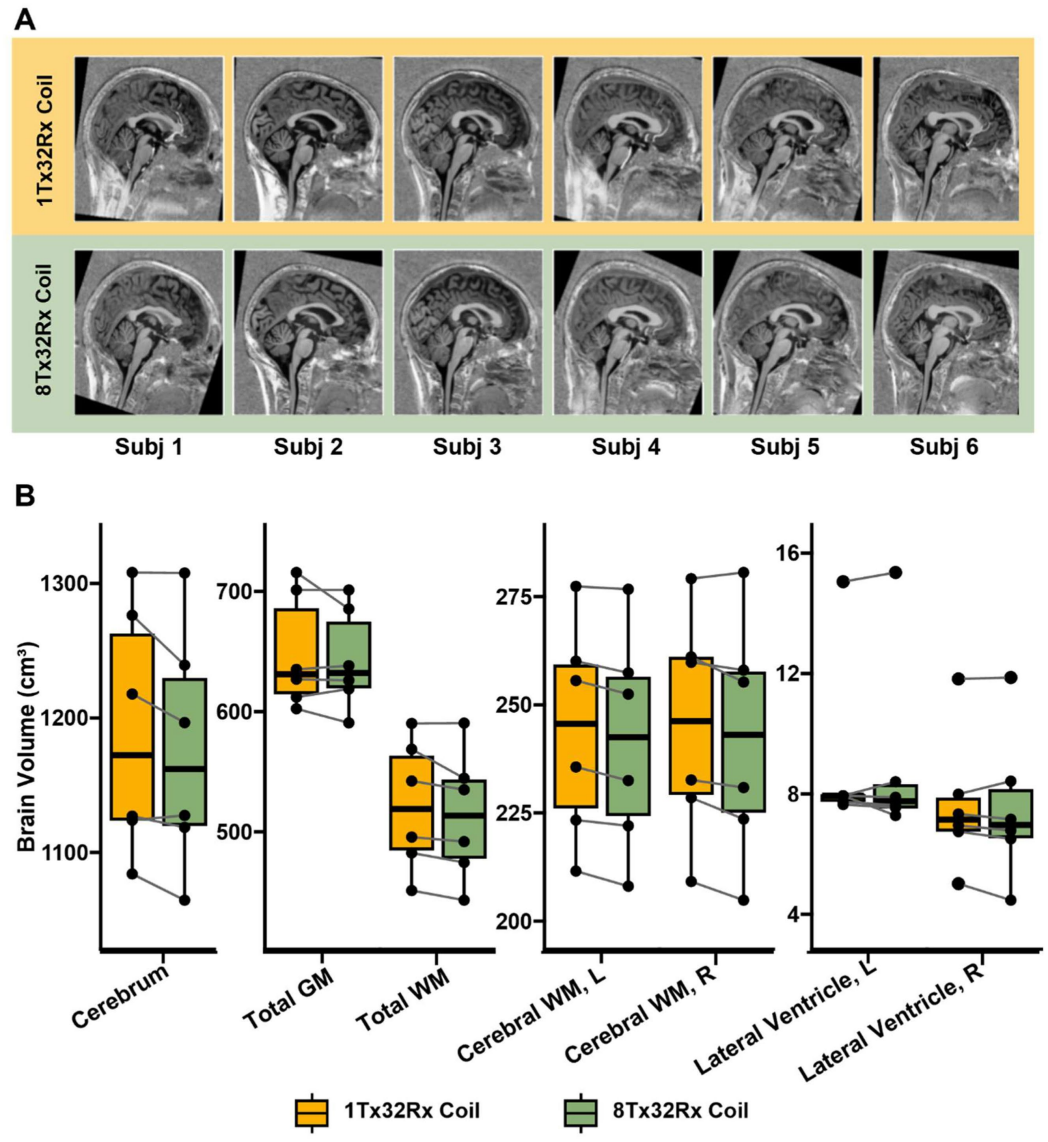
**Structural imaging**. Structural imaging with MP2RAGE **(A)** Sagittal T_1_ weighted MP2RAGE images normalised into the MNI space. On a qualitative level, they provide similar structural detail in the cerebrum, but differ particularly in the inferior brain regions including the cerebellum and cervical spine **(B)** Paired boxplots showing various brain volumes as segmented by FreeSurfer. The measured volumes were comparable across coils (mean difference (range) = −1.3% (−11.6, 5.7%), *p* > 0.16) and all regions of interest.

In a qualitative comparison of the UNI-MP2RAGE, the spinal cord was only defined in 50% of the subjects (subjects 2, 3, 6) with the 1Tx32Rx coil, while clear boundaries between the spinal cord and spinal canal were seen in all volunteers with the 8Tx32Rx coil (Figure 3A). In the cerebrum, both systems showed similar MP2RAGE image quality, with no significant differences in grey, white matter, and ventricular volumes (Figure 3B). However, qualitative differences were seen in the brainstem and cervical spine.

**Figure 4:**
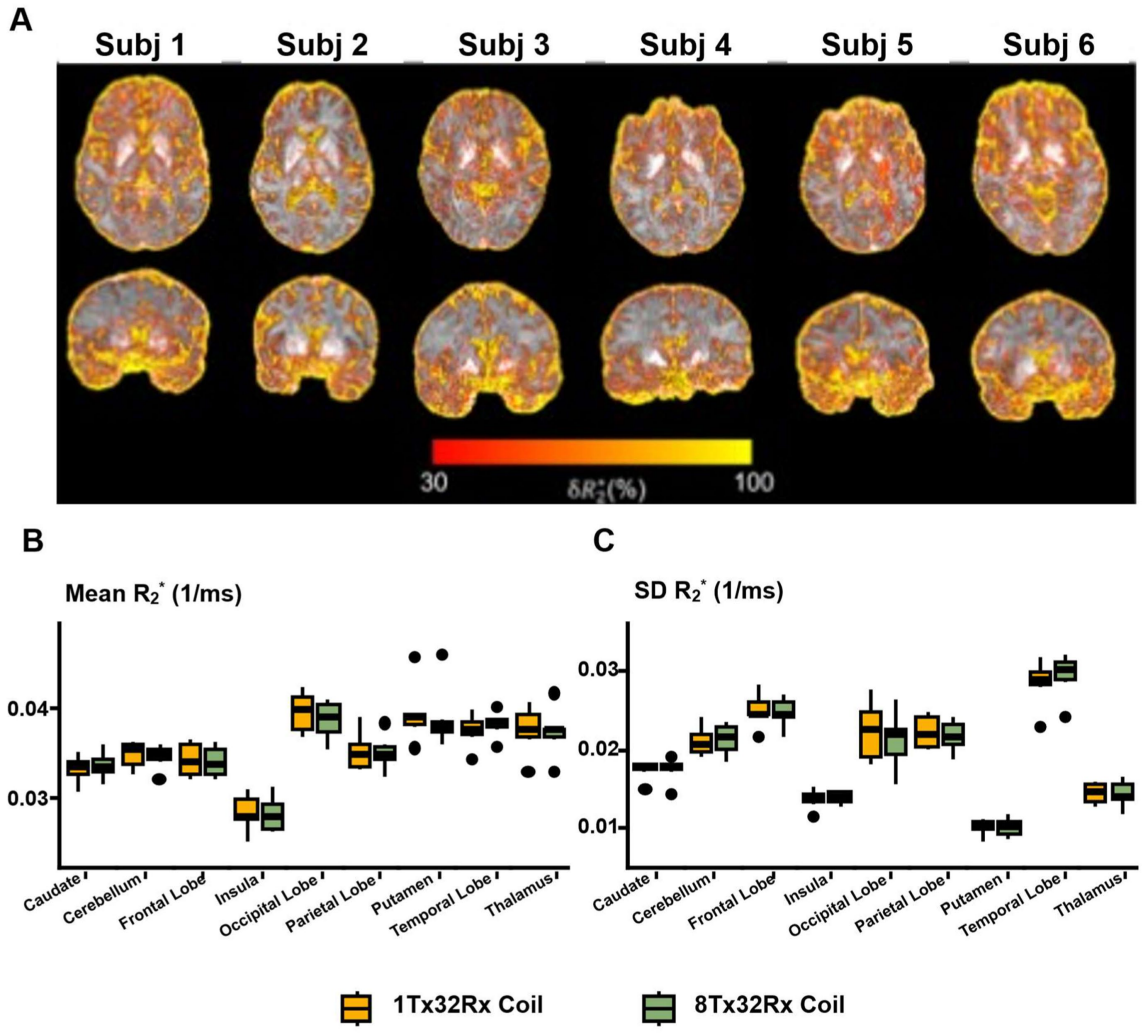
R_2_^*^. **(A)** δR_2_* maps in native image space. **(B, C)** Paired boxplots of means and standard deviations (SD) of R_2_* values within each anatomical ROI. Variability between coils was small across ROIs, with a mean pairwise difference of −0.5% between coils (range = (−8.4, +7.3%), *p* > 0.97). **(C)** Further indicates, that within-ROI-homogeneity of R_2_* was also comparable across coils (mean difference = −0.5%, range = (−15.8, +9.4%), *p* > 0.11).

In terms of R_2_*, differences between the 1Tx32Rx and 8Tx32Rx coils were observed in vessels, edges of the cortex like the cerebral-spinal-fluid (CSF) or near the sinuses (Figure 4A) which are commonly associated with more considerable B_0_ variability. Regions with high tissue susceptibility (e.g., basal ganglia) show reduced δR_2_^*^. On average across subjects, the δR_2_^*^ measured in the CSF was 2.3-fold higher compared to the caudate, insula, putamen and thalamus ROIs. Average R_2_* values extracted from the nine anatomical ROIs were not significantly different across the two systems (Figure 4B) (all pairwise *p*-values > 0.865). Likewise, the standard deviation within these ROIs was consistent (all pairwise *p*-values > 0.951, Figure 4C), demonstrating the interchangeability of the 1Tx32Rx and 8Tx32Rx coils for R_2_* imaging.

**Figure 5:**
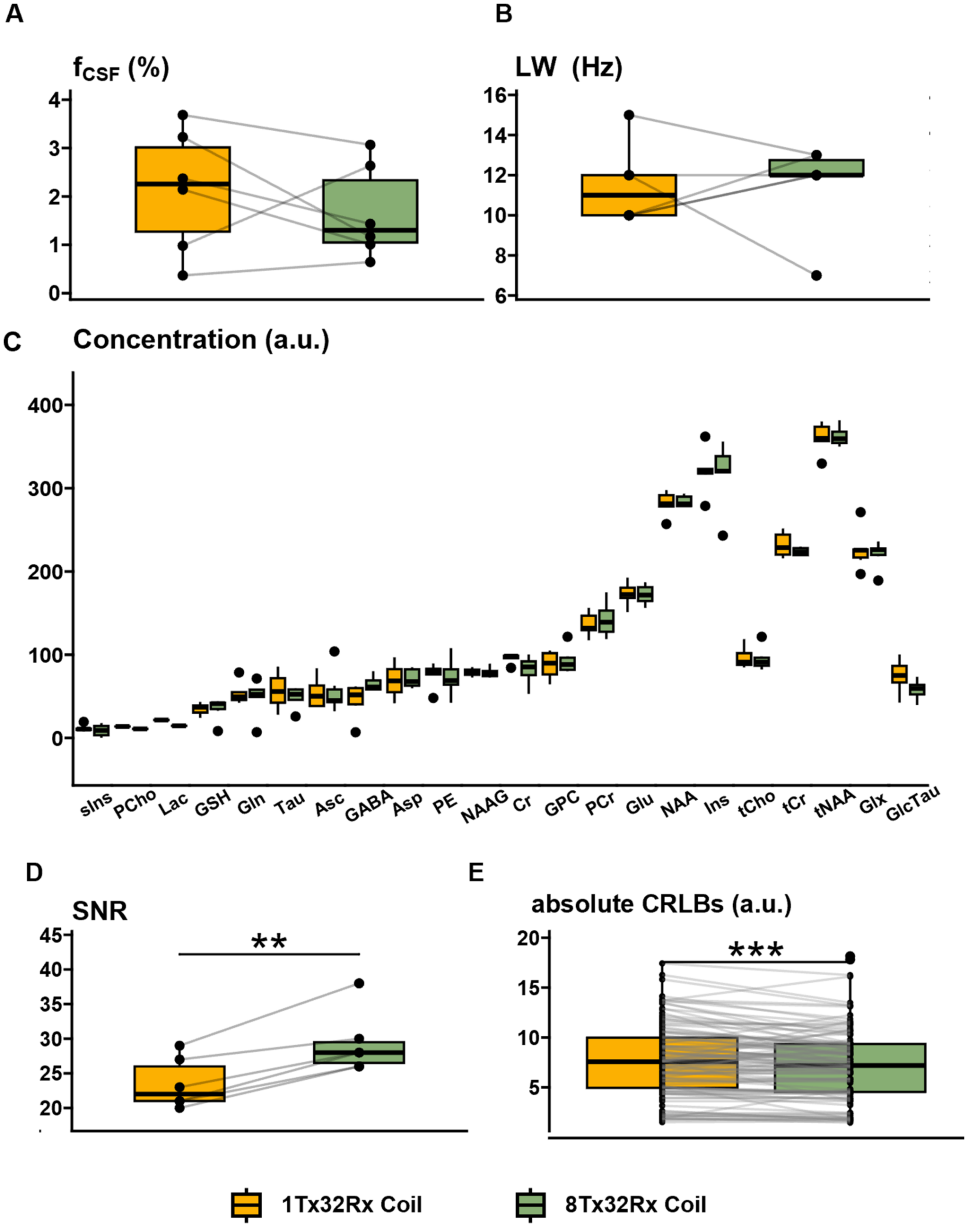
**MRS**. Boxplots of MR spectroscopy quality metrics and fitting results. **(A)** Comparison of percentage CSF volume as an indicator for consistent voxel placement (*p* > 0.05). **(B)** Spectral linewidths were also comparable between coils (*p* > 0.05), indicating similar shimming performance between coils for brainstem MRS. **(C)** Metabolite concentrations for healthy volunteers. After correction for multiple comparisons, none of the metabolite concentrations were significantly different (*p* > 0.56). **(D)** However, the 27% increase of SNR for the 8Tx32Rx coil (*p* < 0.001) may be supporting the lower absolute CRLBs for metabolite concentrations which met minimum individual quality requirements shown in **(E).**

Subjective voxel placement of the spectroscopic VOI was aided by the improved definition of the brainstem and upper cervical cord in the 8Tx32Rx coil (Figure 3A, Supplementary Figure S4). Therefore, the volumetric percentage of CSF in the VOI was consistent between systems (Figure 5A), which aided in comparable shimming performance as indicated by consistent linewidths (Figure 5B). No significant differences in measured metabolite concentrations was found (*p* > 0.15, Figure 5C). However, spectra acquired with the 8Tx32 Rx coil had a median SNR increase of 27% (*p* < 0.01, Figure 5D, Supplementary Figure S5) compared to the 1Tx32Rx coil. This likely explains the lower CRLBs for the 8Tx32Rx scans because CRLB measures fitting uncertainty for individual metabolites and higher SNR data gives lower uncertainty (Figure 5E).

**Figure 6:**
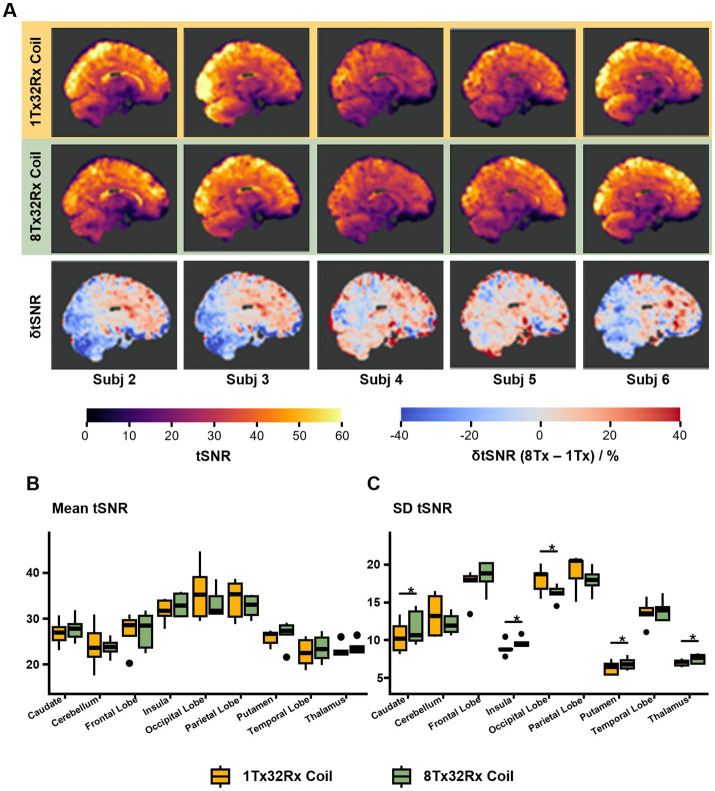
**EPI**. **(A)** Sagittal view of tSNR and δtSNR (8Tx – 1Tx) maps for both coils normalised into MNI space. Coronal and transverse maps are shown in Supplementary Figure S3. **(B)** Box plot showing mean tSNR in the anatomical ROIs defined by the MNI atlas. **(C)** Box plot showing standard deviations in tSNR in the anatomical ROIs defined by the MNI atlas. * = statistically significant difference (*p* < 0.05) between 1Tx32Rx and 8Tx32Rx tSNR value detected, adjusted for multiple comparisons.

Figure 6 shows the GRE-EPI tSNR maps and voxel-wise differences in tSNR (δtSNR) (Figure 6A). The mean and standard deviation of tSNR within each ROI are shown in Figures 6B-C. There were no significant differences in mean tSNR in any ROIs (*p* > 0.53, Table 1, Figure 6B). The 8Tx32Rx coil showed significantly higher local variability in tSNR in four basal-ganglia ROIs (caudate, insula, putamen, thalamus) but significantly lower variations in tSNR in the occipital-lobe ROI (Table 1, Figure 6C). Additional multiplanar tSNR maps are shown in Supplementary Figure S6.

**Figure 7:**
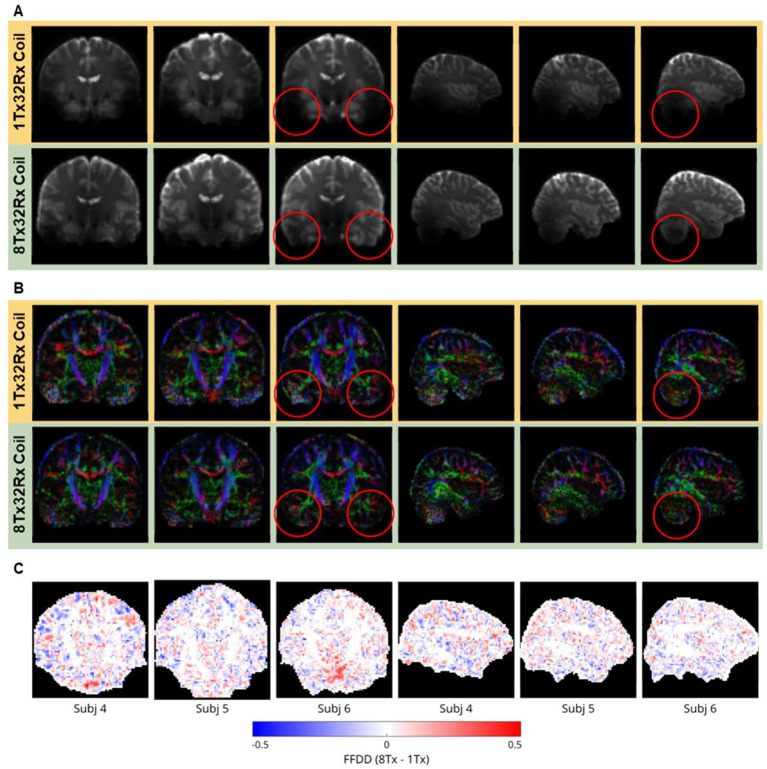
**DTI**. **(A)** Coronal and sagittal view of the spin-echo (non diffusion-weighted, i.e., *b* = 0) images in the native imaging space showing extensive signal dropouts in the temporal lobes and cerebellum with the 1Tx32Rx coil (top row), which is alleviated with the 8Tx32Rx coil (bottom row) and may be driven by the higher B_1_^+^ efficiency in the temporal lobes (Figures 2B,C). Red circles highlight example areas in the temporal lobes and cerebellum where the use of the 8Tx32Rx coil lead to a visual improvement of the DTI data. **(B)** Corresponding direction-encoded fractional anisotropy maps in the native imaging space. **(C)** First Fibre Dispersion Difference (FFDD) maps show largely similar performance between coils across the brain, although statistical analysis revealed significantly lower fibre dispersion for the 8Tx32Rx coil in two participants (Subj 4 and 5).

DTI images from the three participants with DTI data are shown in Figure 7. The b = 0 images (Figure 7A) show extensive signal dropouts in the temporal lobes and cerebellum with the 1Tx32Rx coil, which is partially alleviated with the 8Tx32Rx coil. The increased signal in those areas translates into more clearly defined white matter tracts in the Fractional Anisotropy (FA) maps (Figure 7B). First Fibre Dispersion Difference (FFDD) maps are shown in Figure 7C. The presented slices crossing the middle of the brain are representative for the whole brain volume. Upon visual inspection both coils performed similarly. However, a two-tail Mann–Whitney *U*-test revealed that across the whole brain FFD distributions differed between the coils in two participants (Subj 4 and Subj 5). A one-tailed u-test for these two participants then revealed lower dispersion of the 8Tx32Rx coil data in both cases, with mean dispersion lower by 0.75 and 3%, respectively.

## Discussion

Our data show that routine neuroimaging sequences can be run with comparable results and partially improved data quality on the 8Tx32Rx system compared to the 1Tx32Rx system. Many sites in the ultra-high field community have access to both commercial head coils and as such these coils were selected in various multi-centre imaging harmonisation studies, such as those conducted by the UK7T Network (Clarke et al., 2020), the European Ultrahigh-Field Imaging Network in Neurodegenerative Diseases (EUFIND) (Düzel et al., 2019), and German Ultra-high Field Imaging (GUFI) (Voelker et al., 2016; Voelker et al., 2021).

When operating these two coils in CP mode on the Siemens 7T Terra platform, both coils have the same *k*-factor for local SAR calculations (*k* = 1.00 kg^−1^) (Shirvani et al., 2020). Our data shows that the 8Tx32Rx coil provides better B_1_^+^ efficiency and homogeneity in posterior and inferior brain regions compared to the 1Tx32Rx coil (Figure 2), with otherwise similar or better performance across the common neuroimaging protocols that we have tested (Figures 3–7).

This improved B_1_^+^ has translated into data quality improvements in other imaging sequences, most notably the MP2RAGE, MRS and DTI acquisitions. Specifically, we saw enhanced spinal cord visualization in the cervical spine region in MP2RAGE (Figure 3A). This aided the voxel placement for MRS and might be beneficial for studies in multiple sclerosis where cervical cord atrophy at the C1 and C2/3 levels is a strong indicator of impending conversion from relapsing–remitting multiple sclerosis to secondary progressive multiple sclerosis (Casserly et al., 2018). Similarly, the recovery of DTI signal in the temporal lobes using the 8Tx32Rx coil may be of value for studies using diffusion to investigate aphasia syndromes (Glasser and Rilling, 2008) or temporal lobe epilepsy (Lin et al., 2020). The higher SNR for brainstem MRS may be of interested in studies investigating lower concentrated metabolites that typically suffer from high CRLBs, i.e., Gln & GABA for a more robust quantification.

The GRE-EPI and R_2_^*^ image quality were equivalent for both coils, which suggests that they may be largely interchangeable for these modalities. We suspect that these sequences perform more closely between the coils due to their use of exclusively low flip angle pulses. For research studies which only focus on a specific brain area, such as visual fMRI studies, we note that the 1Tx32Rx head coil may be preferable because it has a stronger B_1_^+^ field in the visual cortex as shown in Figure 2B.

Overall, our study shows that the 1Tx32Rx and 8Tx32Rx coils produce broadly comparable data for common neuroimaging protocols used in our centre. The 8Tx32Rx coil gave slightly higher data quality in MP2RAGE, DTI and ^1^H-MRS in the lower brain. This aligns with recent findings by Huber et al. (2025) who showed that the two coils differ in geometry, challenging the previous assumption of equivalence and introducing uncertainty for resting-state fMRI applications where the coil selection depends on a study’s aims. This information has helped us present evidence-based advice to colleagues who are setting up research studies on the 7T.

Our findings extend the comparisons of Wu et al. (2018, 2019), who compared diffusion and resting-state fMRI acquisitions between the 1Tx32Rx and dynamic multi-band acquisitions on the 8Tx32Rx system. Interestingly, Wu et al. used dielectric padding to improve B_1_^+^ homogeneity in the 1Tx32Rx coil, which can reduce participant compliance due to discomfort and may introduce additional long-term variability due to degradation of the high-permittivity compound. Notably, Wu et al. did not evaluate the performance of the 8Tx32Rx coil operating in CP mode, in contrast to the assessment performed in this present study.

Furthermore, several recent publications have focused on applying pTx for clinical research at 7T, with improved workflows being presented for MPRAGE (Herrler et al., 2021), GRE-EPI for BOLD imaging (Ding et al., 2020), TSE (Malik et al., 2015), FLAIR (Klodowski et al., 2025; Gras et al., 2018), and diffusion imaging (Ding et al., 2022; Williams et al., 2022). Thus, from a technical development perspective, it is attractive to scan with the 8Tx32Rx coil since it opens up the opportunity of including advanced pTx sequences as add-on scans. We caution, however, that coil choice should be carefully considered for layer-fMRI, as recent work highlights coil-dependent differences that may favour the 1Tx32Rx coil in specific use cases (Huber et al., 2025).

In addition, on the next generation of MRI platforms, pTx infrastructure is increasingly becoming the norm, rather than an optional extra (Williams et al., 2023). In this context, the present work is particularly relevant, as systems such as Siemens’ Terra.X are actively transitioning clinical studies to the 8Tx32Rx head coil, and our findings help support the appropriateness of this shift within the risk-averse framework of clinical translational research.

### Limitations

It should be noted that only neuroimaging protocols in regular use at our site were tested. It is recommended that similar comparisons, inclusive of participant samples representative of the local cohorts, be performed at other sites with significantly different local standard protocols including other MRI contrasts or different coils.

While the neuroimaging coils used for this experiment are widely used in the research community, in particular layer fMRI (Huber et al., 2025), there is a plethora of alternative neuroimaging coils available at 7T. Furthermore, the specific coils at our site do not possess CE-marking, which may cause challenges for the implementation into routine clinical scanning. Additionally, the assessment was primarily centred on technical metrics of data quality, providing a solid foundation for the current findings. However, there is potential for future research to expand upon our work by exploring inter- and intra-coil reproducibility, or evaluate other standard neuroimaging sequences thereby offering a more comprehensive understanding for the opportunity of data pooling across different coil types.

## Conclusion

Overall, data quality and quantitative MRI metrics were comparable for standard neuroimaging protocols with both coils. The 8Tx32Rx coil produced higher B_1_^+^ and SNR in MRS in the inferior brain regions, benefiting studies targeting those areas. Running standard neuroimaging protocols on the 8Tx32Rx coil has the benefit of allowing advanced parallel transmit sequences with improved image quality to be added to the protocol with minimal disruption. We believe other sites could usefully investigate migrating their routine neuroimaging protocols to the 8Tx32Rx coil. In this context, the 8Tx32Rx coil should be preferred to the 1Tx32Rx head coil for routine neuroimaging protocols, similar to the one tested in this study.

## Data Availability

The datasets presented in this article are not readily available because of restrictions imposed by our institutions on privacy/ethical grounds. Requests to access the datasets should be directed to the corresponding author.

## Author disclaimer

The views expressed are those of the author(s) and not necessarily those of the National Institute for Health Research (NIHR) or the Department of Health and Social Care. For the purpose of open access, the author has applied a CC BY public copyright licence to any Author Accepted Manuscript version arising from this submission.
