# Supplementary material for: Comparison of commercial 1Tx32Rx vs. 8Tx32Rx head coils for routine 7T neuroimaging
Source: Front Neuroimaging. 2026 Mar 25;5:1736950. doi: 10.3389/fnimg.2026.1736950 (PMC13056848; doi:10.3389/fnimg.2026.1736950)
Supplement: Supplementary file 1 [file Table_1.DOCX]

# Title Page

**Supplemental Material**

# Supplementary Tables

## Supplementary Table S1: Summary of scans performed on each subject.

| **Scan** | **Subject 1** | **Subject 2** | **Subject 3** | **Subject 4** | **Subject 5** | **Subject 6** |
| --- | --- | --- | --- | --- | --- | --- |
| **B­_0_ mapping** | Y, 35 slices | Y, 35 slices | Y, 35 slices | Y, 50 slices | Y, 50 slices | Y, 50 slices |
| **B_1_ mapping** | Y | Y | Y | Y | Y | Y |
| **MP2RAGE** | Y | Y | Y | Y | Y | Y |
| **T_2_^*^** | Y | Y | Y | Y | Y | Y |
| **MRS** | Y | Y | Y | Y | Y | Y |
| **EPI** |  | Y | Y | Y | Y | Y |
| **DTI** |  |  |  | Y | Y | Y |

## **Supplementary Table S2:** MRS Sequence, Processing and Fitting details as per Minimum Reporting Standards in MRS

| **Exam Name** | **Scan A** | **Scan B** |
| --- | --- | --- |
| **1. Hardware** |  |  |
| - 1. Field strength [T] | 7 T | |
| - 1. Manufacturer | Siemens Healthineers, Erlangen, Germany | |
| - 1. Model (software version if available) | Magnetom TERRA 7T (VE12) | Magnetom TERRA 7T (VE12U SP01) |
| - 1. RF coils: nuclei (transmit/receive), number of channels, type, body part | 1Tx/32Rx ^1^H Head Coil, Nova Medical | 8Tx/32Rx ^1^H Head Coil, Nova Medical |
| - 1. Additional hardware | None | |
| **2. Acquisition** |  |  |
| 1. Pulse sequence | sLASER (CMRR package) [1] | |
| 1. Volume of interest (VOI) and its locations | Ponto-medullar junction in brainstem (**Supplementary Figure 1**) | |
| 1. Nominal VOI size [cm³] | [AP, LR, FH] = 12x12x20 mm³ = 2.88 cm^3^ | |
| - 1. Repetition Time (TR), [s]   2. Echo Time (TE) [ms] | 1. TR = 5 s 2. TE = 28 ms | |
| 1. Total number of excitations or acquisitions per spectrum (NA)   Total number of spectra (acquired / in time-series) | *120 acquisitions per spectrum (NA = 120)* | |
| 1. Additional sequence parameters:    1. number of spectral points    2. spectral width in Hz    3. frequency offsets | - N (spectral points) = 2048, - dwell time = 0.167ms - bandwidth = 6.00 kHz - frequency offset for metabolite excitations = -2.0ppm | |
| 1. Water suppression method | - VAPOR [2] - Water suppression Bandwidth = 135Hz | |
| 1. Shimming method 2. reference peak 3. thresholds for “acceptance of shim” chosen | 1. FASTMAP [3] applied to water signal   We aimed for <15Hz linewidth of unsuppressed water peak at the time of scan. Due to the one-off opportunity to scan patients, we did not abort scans or rescan if this was not achieved. | |
| 1. Triggering or motion correction method | None | |

| **3. Data analysis methods and outputs** | |
| --- | --- |
| a. Analysis software | MRSpa (version 1.5g), LCModel (v6.3-1R), FSL (5.0.8) |
| b. Processing steps deviating from quoted reference or product analysis software (vendor, version) | none |
| c. Output measure  (e.g. absolute concentration, institutional units, ratio) Processing steps deviating from quoted reference or product | Fitted metabolite signal amplitudes, *A_met_*, were scaled using the unsuppressed water signal, A_water ref_, corrected for the volumetric fraction of cerebrospinal fluid (CSF) in the voxel, *f_CSF_,* and scaled by a global scaling factor, f_scale,global_. Concentrations are reported in mM.   \| $[met]$ \| $=\frac{1}{1-f_{\mathrm{CSF}}}\cdot f_{scale,global}\cdot\frac{A_{\mathrm{met}}}{A_{water ref}}$ \| Eq. (1) \| \| --- \| --- \| --- \|   Where  $f_{scale,global}=WCONC\times WVIS$ |
| d. Quantification references and assumptions, fitting model assumptions | - Pure water concentration:   - WCONC = 55500 mmol/L [4] - Water visibility in WM:   - WVIS = 0.653 [4]   The CMRR-provided basis set contained 19 simulated metabolite contributions (Ala, Asp, Asc, Cr, GABA, Glc, Gln, Glu, GPC, GSH, Ins, sIns, Lac, PCr, PCho, PE, NAA, NAAG, Tau) and an experimentally measured macromolecular baseline [5]).Our scripts for processing and fitting and our basis set are available on request. |
| **4. Data Quality** | |
| a. Reported variables   - SNR - Linewidth (with reference peaks) | LCModel SNR  LCModel linewidth (LW) |
| b. Data exclusion criteria | LCModel SNR < 20 or LCModel LW > 15 Hz (~0.051ppm)  Additionally, spectra showing ghosting, lipid or motion artefacts during visual inspection were excluded from further analysis.  All 12 spectra passed these quality criteria. |
| c. Quality measures of postprocessing Model fitting (e.g. CRLB, goodness of fit, SD of residual) | Individual metabolite measurements were rejected when their absolute CRLB was larger than 30% of the mean concentration for each metabolite. This threshold was applied, to reduce bias of filtering for lower concentrated metabolites [6]. |
| d. Sample Spectrum | **Supplementary Figure S2** |

# Supplementary Figures

## Supplementary Figure S1


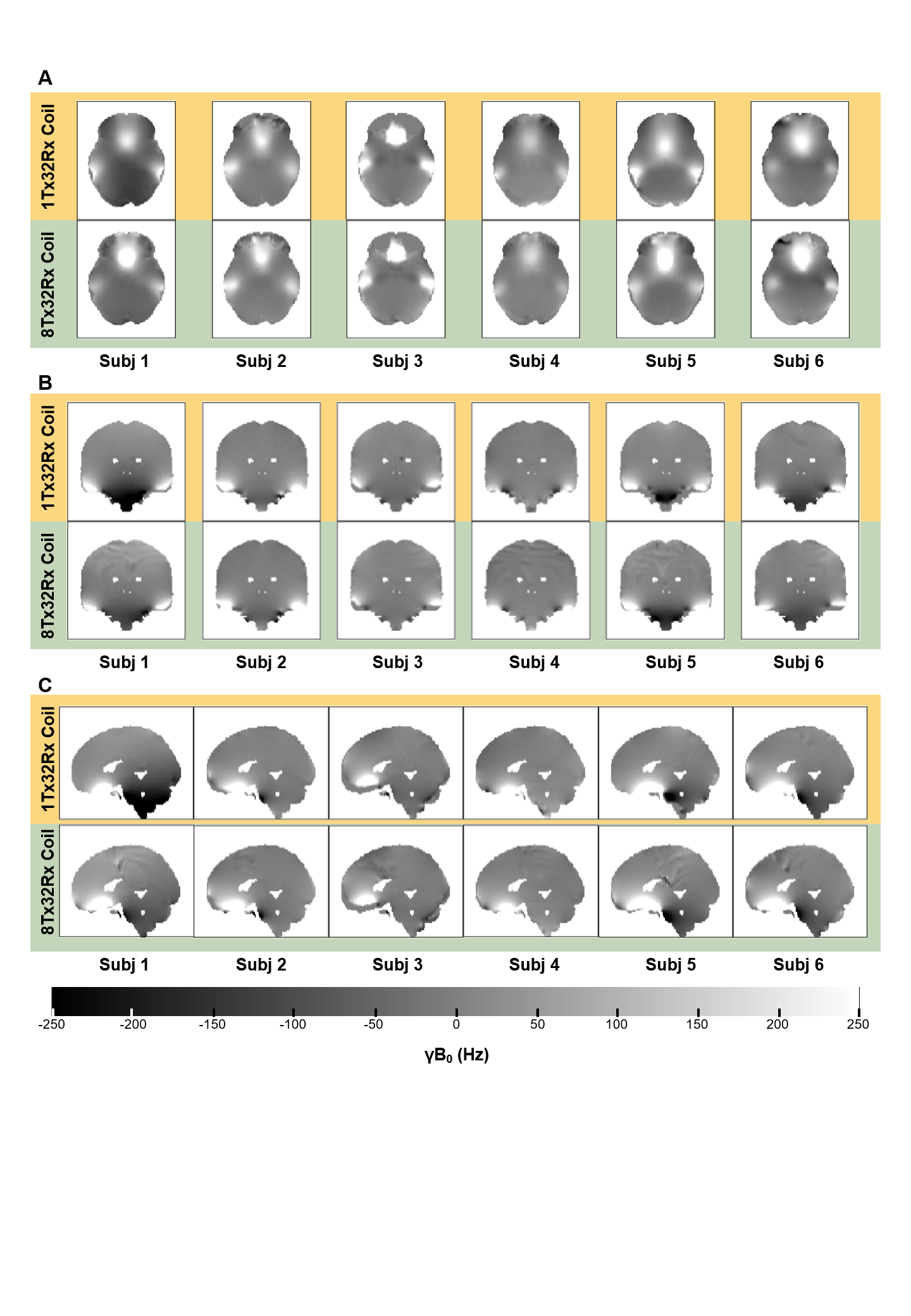


**Supplementary Figure S1** **A** axial**, B** coronal and **C** sagittal B_0_ maps shown as γB_0_ i.e. the local offset from the resonant frequency for all participants registered and brain masked in MNI space.

## Supplementary Figure S2


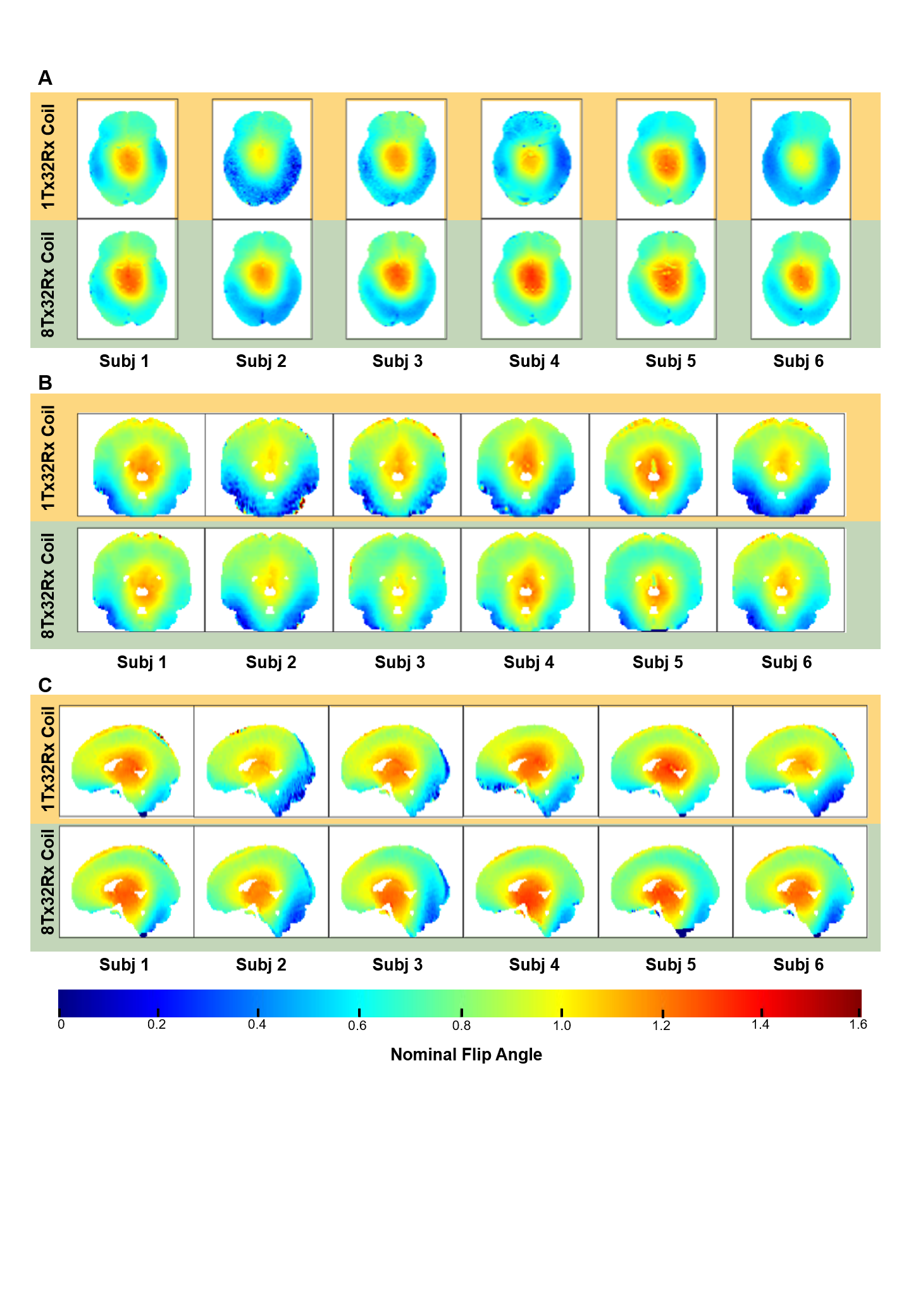


**Supplementary Figure S2 A** axial**, B** coronal and **C** sagittal nominal flip angle maps for all participants registered and masked in MNI space

## Supplementary Figure S3


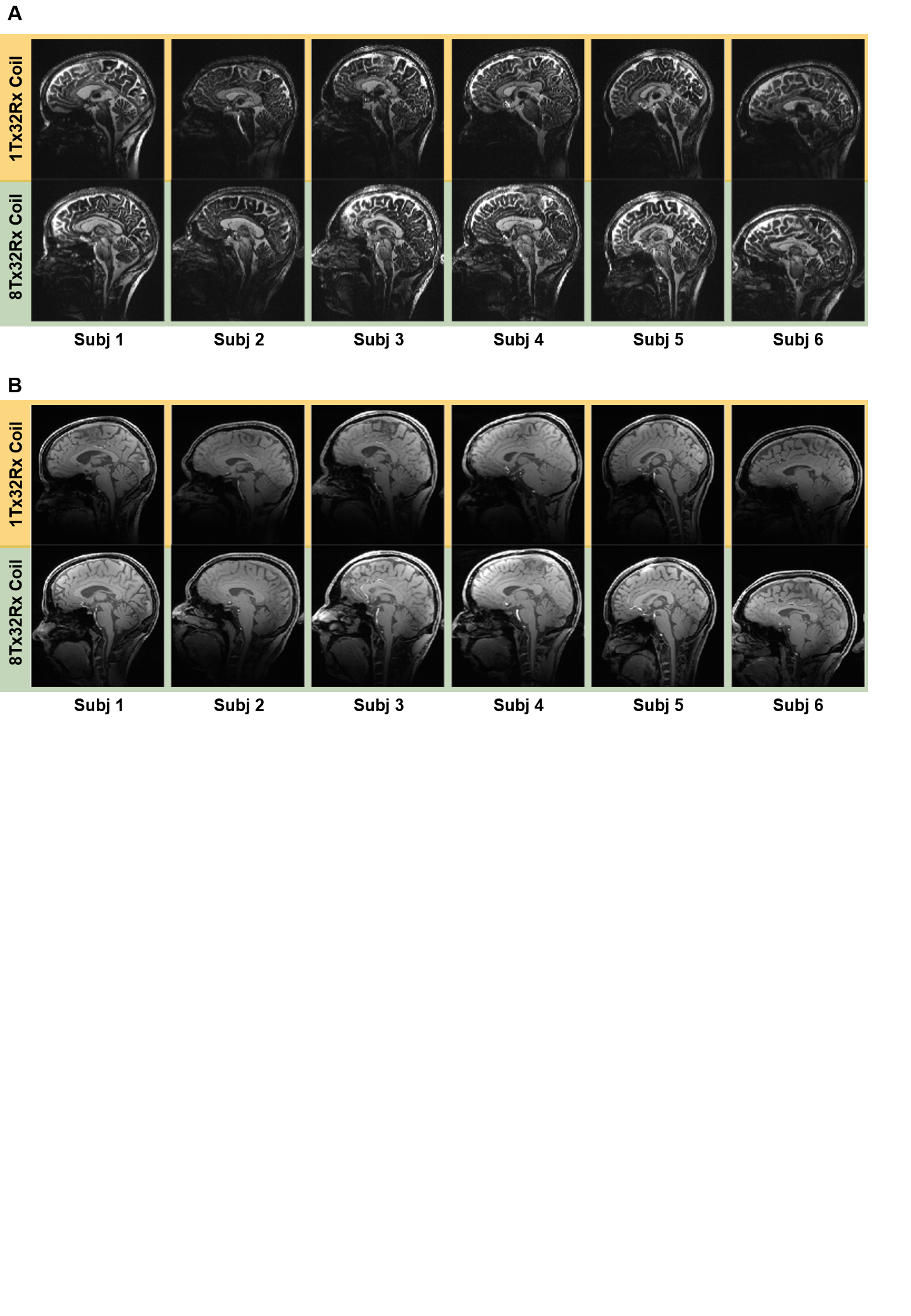


**Supplementary Figure S3 A** 1^st^ (INV1) and **B** 2^nd^ (INV2) inversion images from the MP2RAGE acquisition, highlighting the signal dropouts in the inferior brain regions originate from very low signal in the INV1 image of the 1Tx32Rx coil. The 8Tx32Rx coil provides more details especially around the sinus, cervical spine, and cerebellum. Image windowing is identical across all images.

## Supplementary Figure S4
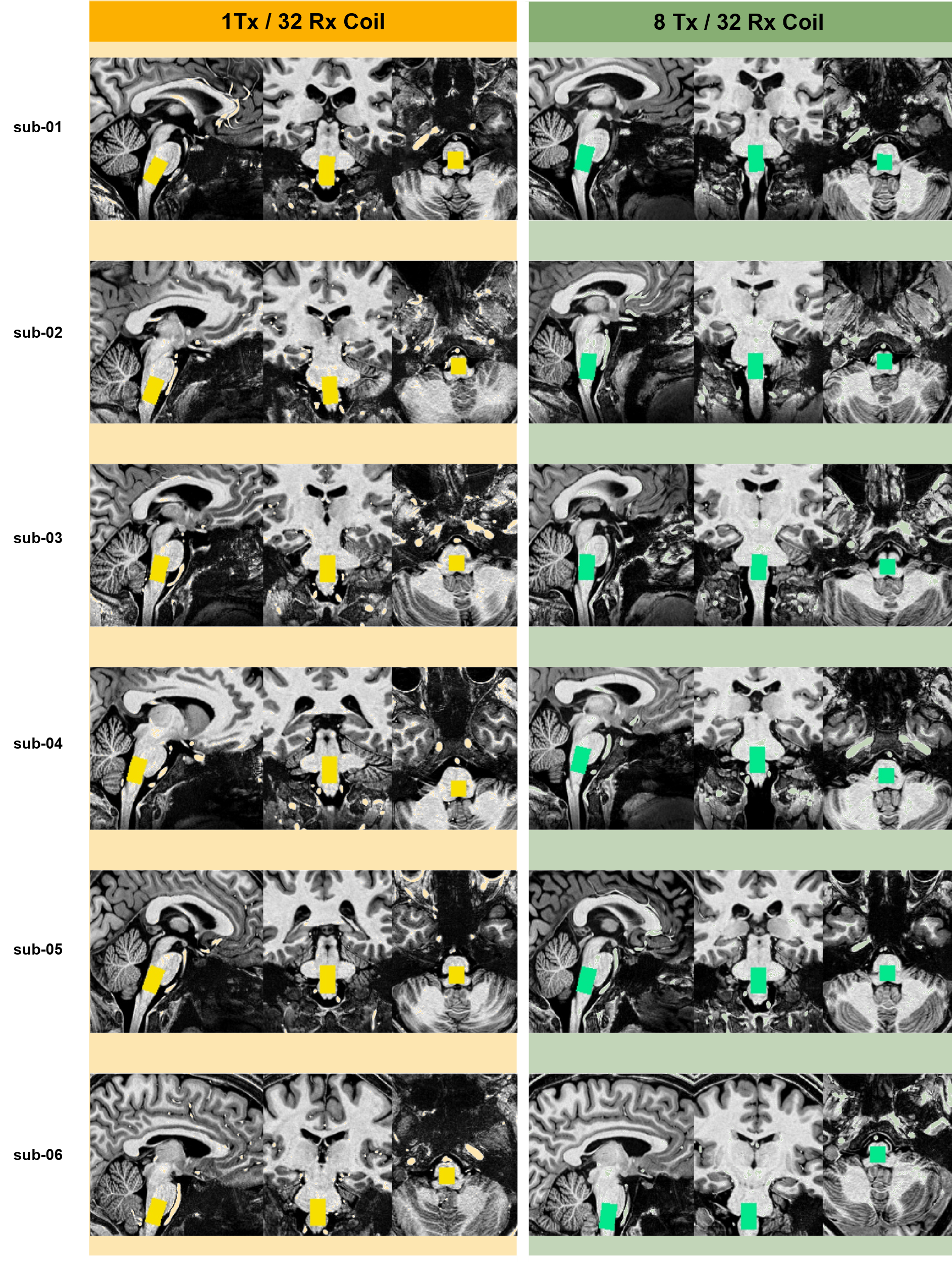


**Supplementary Figure S4** Comparison of voxel placement for ^1^H-MRS on each subject. The voxel was placed centred on the ponto-medullary junction of the brainstem.

## Supplementary Figure S5


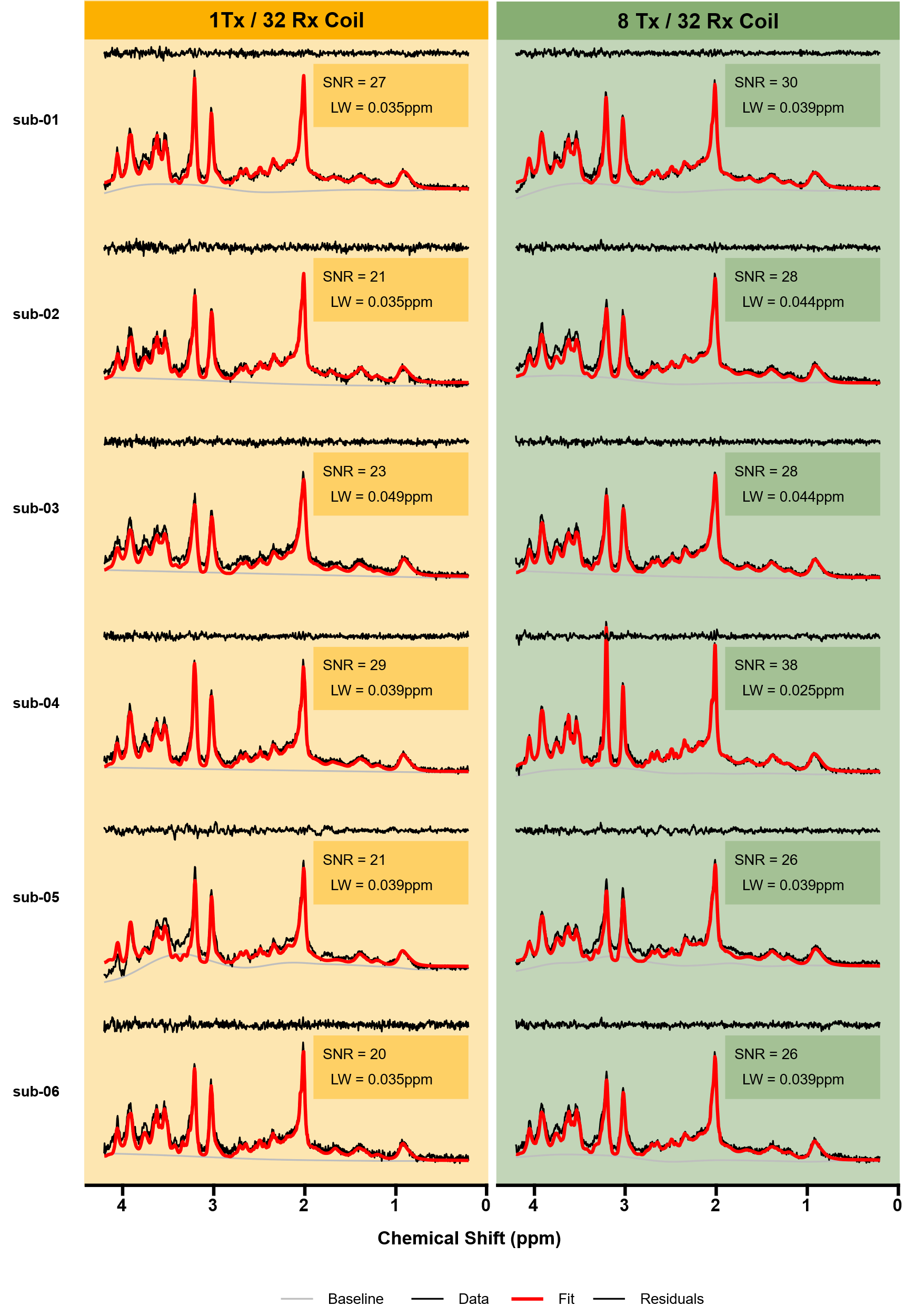


**Supplementary Figure S5** Comparison of spectra obtained with brainstem ^1^H-MRS, including processed spectra (black) after phase correction, frequency alignment and eddy-current correction in MRspa. The LCmodel fit (red) and its estimated baseline (grey) and fit residuals (black) are also shown for comparison. Signal-to-noise ratio (SNR) and LCModel determined linewidths (LW) measured as full-width at half max for each acquisition are included and showed that linewidths were consistent across coils (Table 1 of the main text), indicating reproducible shims. Meanwhile the SNR was significantly higher in the 8Tx/32Rx Coil (mean difference = 5.8, p_corrected_<0.01, 95% CI=(-8.0, -3.7), Table 1).

## Supplementary Figure S6


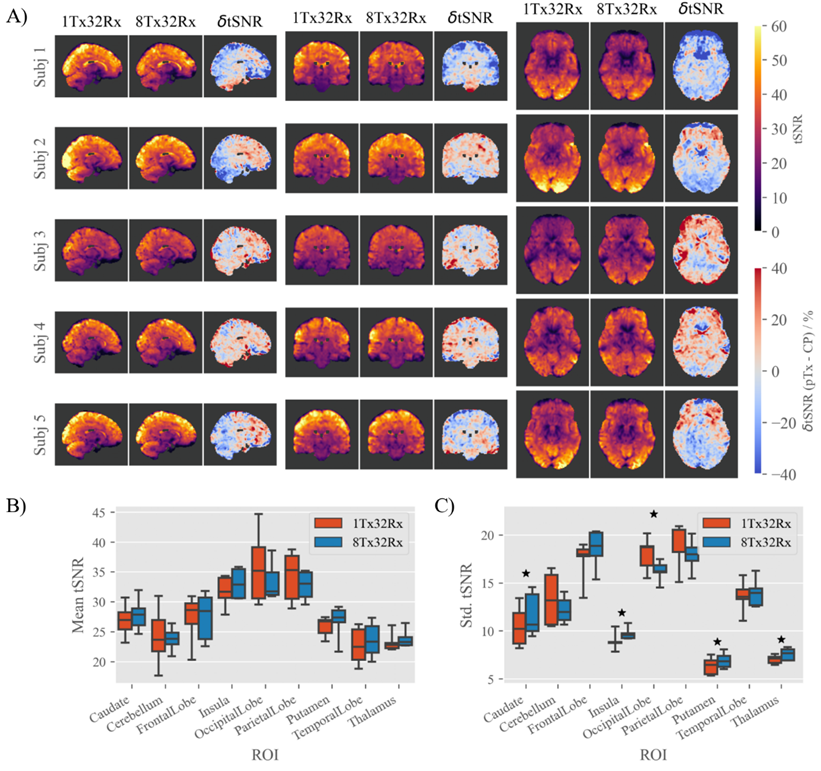


**Supplementary Figure S6*:*** Sagittal, coronal, and transverse view of tSNR and δtSNR (8Tx – 1Tx) maps for both coils normalised into MNI space.

# Supplementary References

1. Oz G, Tkáč I (2011) Short-echo, single-shot, full-intensity proton magnetic resonance spectroscopy for neurochemical profiling at 4 T: validation in the cerebellum and brainstem. Magn Reson Med 65:901–910.

2. Tkác I, Starcuk Z, Choi IY, Gruetter R (1999) In vivo 1H NMR spectroscopy of rat brain at 1 ms echo time. Magn Reson Med 41:649–656.

3. Gruetter R, Tkáč I (2000) Field mapping without reference scan using asymmetric echo-planar techniques. Magn Reson Med 43:319.

4. Gasparovic C, Song T, Devier D, Bockholt HJ, Caprihan A, Mullins PG, Posse S, Jung RE, Morrison LA (2006) Use of tissue water as a concentration reference for proton spectroscopic imaging. Magn Reson Med 55:1219–1226.

5. Terpstra M, Cheong I, Lyu T, Deelchand DK, Emir UE, Bednařík P, Eberly LE, Öz G (2016) Test-retest reproducibility of neurochemical profiles with short-echo, single-voxel MR spectroscopy at 3T and 7T. Magn Reson Med 76:1083–1091.

6. Kreis R (2016) The trouble with quality filtering based on relative Cramér-Rao lower bounds. Magn Reson Med 75:15–18.
