# Supplementary material for: Comparison of commercial 1Tx32Rx vs. 8Tx32Rx head coils for routine 7T neuroimaging
Source: Front Neuroimaging. 2026 Mar 25;5:1736950. doi: 10.3389/fnimg.2026.1736950 (PMC13056848; doi:10.3389/fnimg.2026.1736950)
Supplement: Supplementary file 2 [file Data_Sheet_1.PDF]

gre_b0map_more_slices

TA: 0:54 PM: FIX Voxel size: 2.0×2.0×1.6 mmPAT: 2 Rel. SNR: 1.00 : fm

Properties

Prio recon	Off
Load images to viewer	On
Inline movie	Off
Auto store images	On
Load images to stamp segments	Off
Load images to graphic segments	Off
Auto open inline display	Off
Auto close inline display	Off
Start measurement without further preparation	Off
Wait for user to start	Off
Start measurements	Single measurement

Routine

Slab group	1
Slabs	1
Dist. factor	20 %
Position	L1.0 A22.8 F6.2 mm
Orientation	Transversal
Phase enc. dir.	R >> L
AutoAlign	---
Phase oversampling	0 %
Slice oversampling	0.0 %
Slices per slab	128
FoV read	256 mm
FoV phase	100.0 %
Slice thickness	1.60 mm
TR	5.5 ms
TE 1	1.11 ms
TE 2	3.06 ms
Averages	1
Concatenations	1
Filter	None
Coil elements	A32

Contrast - Common

TR	5.5 ms
TE 1	1.11 ms
TE 2	3.06 ms
MTC	Off
Magn. preparation	None
Flip angle	10 deg
Fat suppr.	None
Water suppr.	None
SWI	Off

Contrast - Dynamic

Averages	1
Averaging mode	Short term
Reconstruction	Magn./Phase
Measurements	1
Multiple series	Off

Resolution - Common

FoV read	256 mm
FoV phase	100.0 %
Slice thickness	1.60 mm
Base resolution	128
Phase resolution	100 %
Slice resolution	100 %

Resolution - Common

Phase partial Fourier	Off
Slice partial Fourier	Off
Interpolation	Off

Resolution - iPAT

PAT mode	GRAPPA
Accel. factor PE	2
Ref. lines PE	24
Accel. factor 3D	1
Reference scan mode	Integrated

Resolution - Filter Image

Image Filter	Off
Distortion Corr.	Off
Prescan Normalize	Off
Normalize	Off
B1 filter	Off

Resolution - Filter Rawdata

Raw filter	Off
Elliptical filter	Off

Geometry - Common

Slab group	1
Slabs	1
Dist. factor	20 %
Position	L1.0 A22.8 F6.2 mm
Orientation	Transversal
Phase enc. dir.	R >> L
Slice oversampling	0.0 %
Slices per slab	128
FoV read	256 mm
FoV phase	100.0 %
Slice thickness	1.60 mm
TR	5.5 ms
Multi-slice mode	Interleaved
Series	Interleaved
Concatenations	1

Geometry - AutoAlign

Slab group	1
Position	L1.0 A22.8 F6.2 mm
Orientation	Transversal
Phase enc. dir.	R >> L
AutoAlign	---
Initial Position	L1.0 A22.8 F6.2
Phase	1.0 mm
Read	-22.8 mm
Shift	-6.2 mm
Initial Rotation	90.00 deg
Initial Orientation	Transversal

Geometry - Saturation

Saturation mode	Standard
Fat suppr.	None
Water suppr.	None
Special sat.	None

Geometry - Tim Planning Suite

Set-n-Go Protocol	Off

Geometry - Tim Planning Suite

Table position	H
Table position	0 mm
Inline Composing	Off

System - Miscellaneous

Positioning mode	FIX
Table position	H
Table position	0 mm
MSMA	S - C - T
Sagittal	R >> L
Coronal	A >> P
Transversal	F >> H
Coil Combine Mode	Sum of Squares
Save uncombined	Off
Matrix Optimization	Off
AutoAlign	---
Coil Select Mode	Default

System - Adjustments

B0 Shim mode	Brain
B1 Shim mode	TrueForm
Confirm freq. adjustment	Off
Assume Dominant Fat	Off
Assume Silicone	Off
Adjustment Tolerance	Auto

System - Adjust Volume

! Position	L1.0 A24.5 H3.9 mm
! Orientation	Transversal
! Rotation	90.00 deg
! R >> L	156 mm
! A >> P	193 mm
! F >> H	155 mm
Reset	Off

System - Tx/Rx

Frequency 1H	297.230405 MHz
Correction factor	1
Gain	High
Img. Scale Cor.	1.000
Reset	Off
? Ref. amplitude 1H	0.000 V

Physio - Signal1

1st Signal/Mode	None
TR	5.5 ms
Concatenations	1
Segments	1

Physio - Cardiac

Tagging	None
Magn. preparation	None
Fat suppr.	None
Dark blood	Off
FoV read	256 mm
FoV phase	100.0 %
Phase resolution	100 %

Physio - PACE

Resp. control	Off
Concatenations	1

Inline - Common

Subtract	Off
Measurements	1
StdDev	Off
Liver registration	Off
Save original images	On

Inline - MIP

MIP-Sag	Off
MIP-Cor	Off
MIP-Tra	Off
MIP-Time	Off
Save original images	On

Inline - Soft Tissue

Wash - In	Off
Wash - Out	Off
TTP	Off
PEI	Off
MIP - time	Off
Measurements	1

Inline - Composing

Inline Composing	Off
Distortion Corr.	Off

Inline - MapIt

Save original images	On
MapIt	None
Flip angle	10 deg
Measurements	1
Contrasts	2
TR	5.5 ms
TE 1	1.11 ms
TE 2	3.06 ms

Sequence - Part 1

Introduction	On
Dimension	3D
Elliptical scanning	Off
Phase stabilisation	Off
Asymmetric echo	Off
Contrasts	2
Flow comp. 1	No
Readout mode	Monopolar
Multi-slice mode	Interleaved
Bandwidth 1	910 Hz/Px
Bandwidth 2	910 Hz/Px

Sequence - Part 2

Segments	1
Acoustic noise reduction	None
RF pulse type	Fast
Gradient mode	Fast
Excitation	Non-sel.
RF spoiling	On

Sequence - Nuclei

TX/RX Nucleus	1H
TX/RX delta frequency	0 Hz
TX Nucleus	None
TX delta frequency	0 Hz
Coil elements	A32

Sequence - Assistant

Mode	Off

b1_mapping_2mm

TA: 0:56 PM: REF Voxel size: 2.0×2.0×2.0 mmPAT: Off Rel. SNR: 1.00 : tfl

Properties

Prio recon	Off
Load images to viewer	On
Inline movie	Off
Auto store images	On
Load images to stamp segments	Off
Load images to graphic segments	Off
Auto open inline display	Off
Auto close inline display	Off
Start measurement without further preparation	Off
Wait for user to start	Off
Start measurements	Single measurement

Routine

Slice group	1
Slices	50
Dist. factor	100 %
Position	L0.2 A3.1 H1.8 mm
Orientation	Transversal
Phase enc. dir.	A >> P
AutoAlign	---
Phase oversampling	0 %
FoV read	220 mm
FoV phase	100.0 %
Slice thickness	2.0 mm
TR	26780.0 ms
TE	2.28 ms
Averages	1
Concatenations	1
Filter	None
Coil elements	VC1

Contrast - Common

TR	26780.0 ms
TE	2.28 ms
Magn. preparation	None
Flip angle	8 deg
Fat suppr.	None
Water suppr.	None

Contrast - Dynamic

Averages	1
Reconstruction	Magnitude
Measurements	1
Multiple series	Each measurement

Resolution - Common

FoV read	220 mm
FoV phase	100.0 %
Slice thickness	2.0 mm
Base resolution	112
Phase resolution	100 %
Phase partial Fourier	Off
Interpolation	Off

Resolution - iPAT

PAT mode	None

Resolution - Filter Image

Image Filter	Off
Distortion Corr.	Off
Prescan Normalize	Off
Normalize	Off
B1 filter	Off

Resolution - Filter Rawdata

Raw filter	Off
Elliptical filter	Off

Geometry - Common

Slice group	1
Slices	50
Dist. factor	100 %
Position	L0.2 A3.1 H1.8 mm
Orientation	Transversal
Phase enc. dir.	A >> P
FoV read	220 mm
FoV phase	100.0 %
Slice thickness	2.0 mm
TR	26780.0 ms
Multi-slice mode	Interleaved
Series	Interleaved
Concatenations	1

Geometry - AutoAlign

Slice group	1
Position	L0.2 A3.1 H1.8 mm
Orientation	Transversal
Phase enc. dir.	A >> P
AutoAlign	---
Initial Position	L0.2 A3.1 H1.8
Phase	-3.1 mm
Read	-0.3 mm
Shift	1.8 mm
Initial Rotation	2.36 deg
Initial Orientation	Transversal

Geometry - Tim Planning Suite

Set-n-Go Protocol	Off
Table position	H
Table position	0 mm
Inline Composing	Off

System - Miscellaneous

Positioning mode	REF
Table position	H
Table position	0 mm
MSMA	S - C - T
Sagittal	R >> L
Coronal	A >> P
Transversal	F >> H
Coil Combine Mode	Sum of Squares
Save uncombined	Off
Matrix Optimization	Off
AutoAlign	---
Coil Select Mode	Default

System - Adjustments

B0 Shim mode	Brain
B1 Shim mode	TrueForm

System - Adjustments

Confirm freq. adjustment	Off
Assume Dominant Fat	Off
Assume Silicone	Off
Adjustment Tolerance	Auto

System - Adjust Volume

Position	L0.2 A3.1 H1.8 mm
Orientation	Transversal
Rotation	2.36 deg
A >> P	220 mm
R >> L	220 mm
F >> H	198 mm
Reset	Off

System - Tx/Rx

Frequency 1H	297.230405 MHz
Correction factor	1
Gain	High
Img. Scale Cor.	1.000
Reset	Off
? Ref. amplitude 1H	0.000 V

Inline - Common

Subtract	Off
Measurements	1
StdDev	Off
Save original images	On

Inline - MIP

MIP-Sag	Off
MIP-Cor	Off
MIP-Tra	Off
MIP-Time	Off
Save original images	On

Inline - Composing

Inline Composing	Off
Distortion Corr.	Off

Sequence - Part 1

Introduction	On
Dimension	2D
Asymmetric echo	Allowed
Flow comp.	No
Multi-slice mode	Interleaved
Echo spacing	4.8 ms
Bandwidth	490 Hz/Px

Sequence - Part 2

RF pulse type	Low SAR
Gradient mode	Normal
Excitation	Slice-sel.
RF spoiling	On
Turbo factor	112

Sequence - Assistant

Mode	Off

mp2rage_sag_p3_0.75mm

TA: 8:50 PM: REF Voxel size: 0.8×0.8×0.8 mmPAT: 3 Rel. SNR: 1.00 : tfl

Properties

Prio recon	Off
Load images to viewer	On
Inline movie	Off
Auto store images	On
Load images to stamp segments	Off
Load images to graphic segments	Off
Auto open inline display	Off
Auto close inline display	Off
Start measurement without further preparation	Off
Wait for user to start	Off
Start measurements	Single measurement

Routine

Slab group	1
Slabs	1
Dist. factor	50 %
Position	L1.0 A37.5 H33.7 mm
Orientation	Sagittal
Phase enc. dir.	A >> P
AutoAlign	Head > Basis
Phase oversampling	0 %
Slice oversampling	0.0 %
Slices per slab	224
FoV read	240 mm
FoV phase	93.8 %
Slice thickness	0.75 mm
TR	4300.0 ms
TE	1.99 ms
Averages	1
Concatenations	1
Filter	None
Coil elements	A32

Contrast - Common

TR	4300.0 ms
TE	1.99 ms
Magn. preparation	Non-sel. IR
TI 1	840 ms
TI 2	2370 ms
Flip angle 1	5.0 deg
Flip angle 2	6.0 deg
Fat suppr.	Water excit. fast
Water suppr.	None

Contrast - Dynamic

Averages	1
Averaging mode	Long term
Reconstruction	Magnitude
Measurements	1
Multiple series	Each measurement

Resolution - Common

FoV read	240 mm
FoV phase	93.8 %
Slice thickness	0.75 mm
Base resolution	320
Phase resolution	100 %
Slice resolution	100 %
Phase partial Fourier	Off

Resolution - Common

Slice partial Fourier	6/8
Interpolation	Off

Resolution - iPAT

PAT mode	GRAPPA
Accel. factor PE	3
Ref. lines PE	36
Accel. factor 3D	1
Reference scan mode	Integrated

Resolution - Filter Image

Image Filter	Off
Distortion Corr.	Off
Prescan Normalize	Off
Normalize	Off
B1 filter	Off

Resolution - Filter Rawdata

Raw filter	Off
Elliptical filter	Off

Geometry - Common

Slab group	1
Slabs	1
Dist. factor	50 %
Position	L1.0 A37.5 H33.7 mm
Orientation	Sagittal
Phase enc. dir.	A >> P
Slice oversampling	0.0 %
Slices per slab	224
FoV read	240 mm
FoV phase	93.8 %
Slice thickness	0.75 mm
TR	4300.0 ms
Multi-slice mode	Single shot
Series	Interleaved
Concatenations	1

Geometry - AutoAlign

Slab group	1
Position	L1.0 A37.5 H33.7 mm
Orientation	Sagittal
Phase enc. dir.	A >> P
AutoAlign	Head > Basis
Initial Position	L1.0 A37.5 H33.7
Phase	-37.5 mm
Read	33.7 mm
Shift	1.0 mm
Initial Rotation	0.00 deg
Initial Orientation	Sagittal

Geometry - Navigator

Geometry - Tim Planning Suite

Set-n-Go Protocol	Off
Table position	H
Table position	0 mm
Inline Composing	Off

System - Miscellaneous

Positioning mode	REF
Table position	H
Table position	0 mm
MSMA	S - C - T
Sagittal	R >> L
Coronal	A >> P
Transversal	F >> H
Coil Combine Mode	Sum of Squares
Save uncombined	Off
Matrix Optimization	Off
AutoAlign	Head > Basis
Coil Select Mode	Default

System - Adjustments

B0 Shim mode	Brain
B1 Shim mode	TrueForm
Confirm freq. adjustment	Off
Assume Dominant Fat	Off
Assume Silicone	Off
Adjustment Tolerance	Auto

System - Adjust Volume

Position	L1.0 A37.5 H33.7 mm
Orientation	Sagittal
Rotation	0.00 deg
A >> P	225 mm
F >> H	240 mm
R >> L	168 mm
Reset	Off

System - Tx/Rx

Frequency 1H	297.230405 MHz
Correction factor	1
Gain	High
Img. Scale Cor.	1.000
Reset	Off
? Ref. amplitude 1H	0.000 V

Physio - Signal1

1st Signal/Mode	None
TR	4300.0 ms
Concatenations	1

Physio - Cardiac

Magn. preparation	Non-sel. IR
TI 1	840 ms
TI 2	2370 ms
Fat suppr.	Water excit. fast
Dark blood	Off
FoV read	240 mm
FoV phase	93.8 %
Phase resolution	100 %

Physio - PACE

Resp. control	Off
Concatenations	1

Inline - Common

Subtract	Off
Measurements	1
StdDev	Off
Save original images	On

Inline - MIP

MIP-Sag	Off
MIP-Cor	Off
MIP-Tra	Off
MIP-Time	Off
Save original images	On

Inline - Composing

Inline Composing	Off
Distortion Corr.	Off

Inline - MapIt

Save original images	On
MapIt	T1 map
Flip angle 1	5.0 deg
Flip angle 2	6.0 deg
Measurements	1
TR	4300.0 ms
TE	1.99 ms

Sequence - Part 1

Introduction	On
Dimension	3D
Elliptical scanning	Off
Reordering	Linear
Asymmetric echo	Allowed
Flow comp.	No
Multi-slice mode	Single shot
Echo spacing	6.4 ms
Bandwidth	250 Hz/Px

Sequence - Part 2

RF pulse type	Fast
Gradient mode	Fast*
Excitation	Non-sel.
RF spoiling	On
Incr. Gradient spoiling	Off
Turbo factor	168

Sequence - Nuclei

TX/RX Nucleus	1H
TX/RX delta frequency	0 Hz
TX Nucleus	None
TX delta frequency	0 Hz
Coil elements	A32

Sequence - Assistant

Mode	Off

**ep2d_diff_3weights_6dir**
TA: 5:10 PM: FIX Voxel size: 2.0×2.0×2.0 mmPAT: 3 Rel. SNR: 1.00 : epse

Properties

Prio recon	Off
Load images to viewer	On
Inline movie	Off
Auto store images	On
Load images to stamp segments	Off
Load images to graphic segments	Off
Auto open inline display	Off
Auto close inline display	Off
Start measurement without further preparation	Off
Wait for user to start	Off
Start measurements	Single measurement

Routine

Slice group	1
Slices	83
Dist. factor	0 %
Position	L0.1 A32.1 H32.0 mm
Orientation	T > S-2.4 > C0.1
Phase enc. dir.	A >> P
AutoAlign	---
Phase oversampling	0 %
FoV read	220 mm
FoV phase	100.0 %
Slice thickness	2.0 mm
TR	7000 ms
TE	41.0 ms
Concatenations	1
Filter	Prescan Normalize
Coil elements	A32

Contrast - Common

TR	7000 ms
TE	41.0 ms
MTC	Off
Magn. preparation	None
Flip angle exc	90 deg
Flip angle fat sat	110 deg
Fat suppr.	Fat sat.
Fat sat. mode	Weak

Contrast - Dynamic

Averaging mode	Long term
Reconstruction	Magnitude
Measurements	1
Delay in TR	0 ms
Multiple series	Off

Resolution - Common

FoV read	220 mm
FoV phase	100.0 %
Slice thickness	2.0 mm
Base resolution	110
Phase resolution	100 %
Phase partial Fourier	6/8
Interpolation	Off

Resolution - iPAT

Accel. mode	GRAPPA
Accel. factor PE	3
Ref. lines PE	57
Reference scan mode	EPI/separate

Resolution - Filter Image

Distortion Corr.	Off
Prescan Normalize	On
Dynamic Field Corr.	Off

Resolution - Filter Rawdata

Raw filter	Off
Elliptical filter	Off

Geometry - Common

Slice group	1
Slices	83
Dist. factor	0 %
Position	L0.1 A32.1 H32.0 mm
Orientation	T > S-2.4 > C0.1
Phase enc. dir.	A >> P
FoV read	220 mm
FoV phase	100.0 %
Slice thickness	2.0 mm
TR	7000 ms
Multi-slice mode	Interleaved
Series	Interleaved
Concatenations	1

Geometry - AutoAlign

Slice group	1
Position	L0.1 A32.1 H32.0 mm
Orientation	T > S-2.4 > C0.1
Phase enc. dir.	A >> P
AutoAlign	---
Initial Position	L0.1 A32.1 H32.0
Phase	-32.0 mm
Read	1.2 mm
Shift	32.0 mm
Initial Rotation	0.00 deg
Initial Orientation	T > S
T > S	-2.4
> C	0.1

Geometry - Saturation

Fat suppr.	Fat sat.
Fat sat. mode	Weak
Special sat.	None

Geometry - Navigator

Geometry - Tim Planning Suite

Set-n-Go Protocol	Off
Table position	H
Table position	0 mm
Inline Composing	Off

System - Miscellaneous

Positioning mode	FIX
Table position	H
Table position	0 mm

System - Miscellaneous

MSMA	S - C - T
Sagittal	R >> L
Coronal	A >> P
Transversal	F >> H
Coil Combine Mode	Adaptive Combine
Matrix Optimization	Off
AutoAlign	---
Coil Select Mode	Default

System - Adjustments

B0 Shim mode	Standard
B1 Shim mode	TrueForm
Confirm freq. adjustment	Off
Assume Dominant Fat	Off
Assume Silicone	Off
Adjustment Tolerance	Auto

System - Adjust Volume

Position	L0.1 A32.1 H32.0 mm
Orientation	T > S-2.4 > C0.1
Rotation	0.00 deg
A >> P	220 mm
R >> L	220 mm
F >> H	166 mm
Reset	Off

System - Tx/Rx

Frequency 1H	297.230405 MHz
Correction factor	1
Gain	High
Img. Scale Cor.	1.000
Reset	Off
? Ref. amplitude 1H	0.000 V

Physio - Signal1

1st Signal/Mode	None
TR	7000 ms
Concatenations	1

Physio - PACE

Resp. control	Off
Concatenations	1

Diff - Neuro

Diffusion mode	MDDW
Diff. directions	6
Diffusion Scheme	Monopolar
Diff. weightings	3
b-value 1	0 s/mm²
b-value 2	500 s/mm²
b-value 3	1000 s/mm²
b-value 1	3
b-value 2	3
b-value 3	3
Diff. weighted images	On
Trace weighted images	On
ADC maps	On
FA maps	On
Mosaic	On
Tensor	On
Noise level	30

Diff - Body

Diffusion mode	MDDW
Diff. directions	6
Diffusion Scheme	Monopolar
Diff. weightings	3
b-value 1	0 s/mm²
b-value 2	500 s/mm²
b-value 3	1000 s/mm²
b-value 1	3
b-value 2	3
b-value 3	3
Diff. weighted images	On
Trace weighted images	On
ADC maps	On
Exponential ADC Maps	Off
FA maps	On
Invert Gray Scale	Off
Calculated Image	Off
b-Value >=	0 s/mm²
Noise level	30

Diff - Composing

Inline Composing	Off
Distortion Corr.	Off

Sequence - Part 1

Introduction	On
Optimization	None
Multi-slice mode	Interleaved
Free echo spacing	Off
Echo spacing	0.53 ms
Bandwidth	2674 Hz/Px

Sequence - Part 2

EPI factor	110
RF pulse type	Normal
Gradient mode	Fast

ep2d_diff_3weights_6dir_PA

TA: 0:58 PM: FIX Voxel size: 2.0×2.0×2.0 mmPAT: 3 Rel. SNR: 1.00 : epse

Properties

Prio recon	Off
Load images to viewer	On
Inline movie	Off
Auto store images	On
Load images to stamp segments	Off
Load images to graphic segments	Off
Auto open inline display	Off
Auto close inline display	Off
Start measurement without further preparation	Off
Wait for user to start	Off
Start measurements	Single measurement

Routine

Slice group	1
Slices	83
Dist. factor	0 %
Position	L0.1 A32.1 H32.0 mm
Orientation	T > S-2.4 > C0.1
Phase enc. dir.	P >> A
AutoAlign	---
Phase oversampling	0 %
FoV read	220 mm
FoV phase	100.0 %
Slice thickness	2.0 mm
TR	7000 ms
TE	41.0 ms
Concatenations	1
Filter	Prescan Normalize
Coil elements	A32

Contrast - Common

TR	7000 ms
TE	41.0 ms
MTC	Off
Magn. preparation	None
Flip angle exc	90 deg
Flip angle fat sat	110 deg
Fat suppr.	Fat sat.
Fat sat. mode	Weak

Contrast - Dynamic

Averaging mode	Long term
Reconstruction	Magnitude
Measurements	1
Delay in TR	0 ms
Multiple series	Off

Resolution - Common

FoV read	220 mm
FoV phase	100.0 %
Slice thickness	2.0 mm
Base resolution	110
Phase resolution	100 %
Phase partial Fourier	6/8
Interpolation	Off

Resolution - iPAT

Accel. mode	GRAPPA
Accel. factor PE	3

Resolution - iPAT

Ref. lines PE	57
Reference scan mode	EPI/separate

Resolution - Filter Image

Distortion Corr.	Off
Prescan Normalize	On
Dynamic Field Corr.	Off

Resolution - Filter Rawdata

Raw filter	Off
Elliptical filter	Off

Geometry - Common

Slice group	1
Slices	83
Dist. factor	0 %
Position	L0.1 A32.1 H32.0 mm
Orientation	T > S-2.4 > C0.1
Phase enc. dir.	P >> A
FoV read	220 mm
FoV phase	100.0 %
Slice thickness	2.0 mm
TR	7000 ms
Multi-slice mode	Interleaved
Series	Interleaved
Concatenations	1

Geometry - AutoAlign

Slice group	1
Position	L0.1 A32.1 H32.0 mm
Orientation	T > S-2.4 > C0.1
Phase enc. dir.	P >> A
AutoAlign	---
Initial Position	L0.1 A32.1 H32.0
Phase	32.0 mm
Read	-1.2 mm
Shift	32.0 mm
Initial Rotation	-180.00 deg
Initial Orientation	T > S
T > S	-2.4
> C	0.1

Geometry - Saturation

Fat suppr.	Fat sat.
Fat sat. mode	Weak
Special sat.	None

Geometry - Navigator

Geometry - Tim Planning Suite

Set-n-Go Protocol	Off
Table position	H
Table position	0 mm
Inline Composing	Off

System - Miscellaneous

Positioning mode	FIX
Table position	H
Table position	0 mm

System - Miscellaneous

MSMA	S - C - T
Sagittal	R >> L
Coronal	A >> P
Transversal	F >> H
Coil Combine Mode	Adaptive Combine
Matrix Optimization	Off
AutoAlign	---
Coil Select Mode	Default

System - Adjustments

B0 Shim mode	Standard
B1 Shim mode	TrueForm
Confirm freq. adjustment	Off
Assume Dominant Fat	Off
Assume Silicone	Off
Adjustment Tolerance	Auto

System - Adjust Volume

Position	L0.1 A32.1 H32.0 mm
Orientation	T > S-2.4 > C0.1
Rotation	-180.00 deg
A >> P	220 mm
R >> L	220 mm
F >> H	166 mm
Reset	Off

System - Tx/Rx

Frequency 1H	297.230405 MHz
Correction factor	1
Gain	High
Img. Scale Cor.	1.000
Reset	Off
? Ref. amplitude 1H	0.000 V

Physio - Signal1

1st Signal/Mode	None
TR	7000 ms
Concatenations	1

Physio - PACE

Resp. control	Off
Concatenations	1

Diff - Neuro

Diffusion mode	MDDW
Diff. directions	6
Diffusion Scheme	Monopolar
Diff. weightings	1
b-value	0 s/mm²
b-value	3
Diff. weighted images	On
Trace weighted images	Off
ADC maps	Off
FA maps	Off
Mosaic	Off
Tensor	Off
Noise level	30

Diff - Body

Diffusion mode	MDDW
Diff. directions	6
Diffusion Scheme	Monopolar
Diff. weightings	1

Diff - Body

b-value	0 s/mm²
b-value	3
Diff. weighted images	On
Trace weighted images	Off
ADC maps	Off
Exponential ADC Maps	Off
FA maps	Off
Invert Gray Scale	Off
Calculated Image	Off
b-Value >=	0 s/mm²
Noise level	30

Diff - Composing

Inline Composing	Off
Distortion Corr.	Off

Sequence - Part 1

Introduction	On
Optimization	None
Multi-slice mode	Interleaved
Free echo spacing	Off
Echo spacing	0.53 ms
Bandwidth	2674 Hz/Px

Sequence - Part 2

EPI factor	110
RF pulse type	Normal
Gradient mode	Fast

T2star_0.7_4echo
TA: 6:55 PM: FIX Voxel size: 0.7×0.7×0.7 mmPAT: 4 Rel. SNR: 1.00 : fl_r

Properties

Prio recon	Off
Load images to viewer	On
Inline movie	Off
Auto store images	On
Load images to stamp segments	Off
Load images to graphic segments	Off
Auto open inline display	Off
Auto close inline display	Off
Start measurement without further preparation	Off
Wait for user to start	Off
Start measurements	Single measurement

Routine

Slab group	1
Slabs	1
Dist. factor	20 %
Position	L3.0 A8.5 H15.1 mm
Orientation	Transversal
Phase enc. dir.	R >> L
AutoAlign	---
Phase oversampling	0 %
Slice oversampling	7.1 %
Slices per slab	224
FoV read	224 mm
FoV phase	87.5 %
Slice thickness	0.70 mm
TR	27.0 ms
TE 1	4.54 ms
TE 2	7.76 ms
TE 3	10.98 ms
TE 4	14.20 ms
Averages	1
Concatenations	1
Filter	None
Coil elements	A32

Contrast - Common

TR	27.0 ms
TE 1	4.54 ms
TE 2	7.76 ms
TE 3	10.98 ms
TE 4	14.20 ms
MTC	Off
Magn. preparation	None
Flip angle	15 deg
Fat suppr.	None
Water suppr.	None
SWI	Off

Contrast - Dynamic

Averages	1
Averaging mode	Short term
Reconstruction	Magn./Phase
Measurements	1
Multiple series	Each measurement

Resolution - Common

FoV read	224 mm
FoV phase	87.5 %

Resolution - Common

Slice thickness	0.70 mm
Base resolution	320
Phase resolution	100 %
Slice resolution	100 %
Phase partial Fourier	Off
Slice partial Fourier	7/8
Interpolation	Off

Resolution - iPAT

PAT mode	GRAPPA
Accel. factor PE	2
Ref. lines PE	40
Accel. factor 3D	2
Ref. lines 3D	24
Reference scan mode	Integrated

Resolution - Filter Image

Image Filter	Off
Distortion Corr.	Off
Prescan Normalize	Off
Normalize	Off
B1 filter	Off

Resolution - Filter Rawdata

Raw filter	Off
Elliptical filter	Off

Geometry - Common

Slab group	1
Slabs	1
Dist. factor	20 %
Position	L3.0 A8.5 H15.1 mm
Orientation	Transversal
Phase enc. dir.	R >> L
Slice oversampling	7.1 %
Slices per slab	224
FoV read	224 mm
FoV phase	87.5 %
Slice thickness	0.70 mm
TR	27.0 ms
Multi-slice mode	Interleaved
Series	Interleaved
Concatenations	1

Geometry - AutoAlign

Slab group	1
Position	L3.0 A8.5 H15.1 mm
Orientation	Transversal
Phase enc. dir.	R >> L
AutoAlign	---
Initial Position	L3.0 A8.5 H15.1
Phase	3.0 mm
Read	-8.5 mm
Shift	15.1 mm
Initial Rotation	90.00 deg
Initial Orientation	Transversal

Geometry - Saturation

Saturation mode	Standard
Fat suppr.	None

Geometry - Saturation

Water suppr.	None
Special sat.	None

Geometry - Tim Planning Suite

Set-n-Go Protocol	Off
Table position	H
Table position	0 mm
Inline Composing	Off

Geometry - Tim CT

Tim CT mode	Off
Slabs	1
Slices per slab	224
Slice thickness	0.70 mm
Dist. factor	20 %
FoV read	224 mm
FoV phase	87.5 %
Segments	1

System - Miscellaneous

Positioning mode	FIX
Table position	F
Table position	0 mm
MSMA	S - C - T
Sagittal	R >> L
Coronal	A >> P
Transversal	F >> H
Coil Combine Mode	Sum of Squares
Save uncombined	Off
Matrix Optimization	Off
AutoAlign	---
Coil Select Mode	Default

System - Adjustments

B0 Shim mode	Brain
B1 Shim mode	TrueForm
Confirm freq. adjustment	Off
Assume Dominant Fat	Off
Assume Silicone	Off
Adjustment Tolerance	Auto

System - Adjust Volume

! Position	Isocenter
! Orientation	Transversal
! Rotation	0.00 deg
! A >> P	192 mm
! R >> L	192 mm
! F >> H	144 mm
Reset	Off

System - Tx/Rx

Frequency 1H	297.230405 MHz
Correction factor	1
Gain	High
Img. Scale Cor.	1.000
Reset	Off
? Ref. amplitude 1H	0.000 V

Physio - Signal1

1st Signal/Mode	None
TR	27.0 ms
Concatenations	1
Segments	1

Physio - Cardiac

Tagging	None
Magn. preparation	None
Fat suppr.	None
Dark blood	Off
FoV read	224 mm
FoV phase	87.5 %
Phase resolution	100 %

Physio - PACE

Resp. control	Off
Concatenations	1

Inline - Common

Subtract	Off
Measurements	1
StdDev	Off
Liver registration	Off
Save original images	On

Inline - MIP

MIP-Sag	Off
MIP-Cor	Off
MIP-Tra	Off
MIP-Time	Off
Save original images	On

Inline - Soft Tissue

Wash - In	Off
Wash - Out	Off
TTP	Off
PEI	Off
MIP - time	Off
Measurements	1

Inline - Composing

Inline Composing	Off
Distortion Corr.	Off

Inline - MapIt

Save original images	On
MapIt	None
Flip angle	15 deg
Measurements	1
Contrasts	4
TR	27.0 ms
TE 1	4.54 ms
TE 2	7.76 ms
TE 3	10.98 ms
TE 4	14.20 ms

Sequence - Part 1

Introduction	On
Dimension	3D
Elliptical scanning	Off
Phase stabilisation	Off
Asymmetric echo	Allowed
Contrasts	4
Flow comp. 1	Yes
Readout mode	Monopolar
Multi-slice mode	Interleaved
Bandwidth 1	430 Hz/Px
Bandwidth 2	430 Hz/Px
Bandwidth 3	430 Hz/Px

Sequence - Part 1

Bandwidth 4	430 Hz/Px

Sequence - Part 2

Segments	1
Acoustic noise reduction	None
RF pulse type	Fast
Gradient mode	Normal
Excitation	Slab-sel.
RF spoiling	On

Sequence - Special

Use WTC SENSE Recon	Off

Sequence - Assistant

Mode	Off

cmrr_mbep2d_bold_3x2_1.5iso_5vols_PA

TA: 0:35 PM: FIX Voxel size: 1.5×1.5×1.5 mmPAT: 2 Rel. SNR: 1.00 : epfid

Properties

Prio recon	Off
Load images to viewer	On
Inline movie	Off
Auto store images	On
Load images to stamp segments	Off
Load images to graphic segments	Off
Auto open inline display	Off
Auto close inline display	Off
Start measurement without further preparation	Off
Wait for user to start	Off
Start measurements	Single measurement

Routine

Slice group	1
Slices	96
Dist. factor	0 %
Position	L3.2 A34.5 H8.8 mm
Orientation	Transversal
Phase enc. dir.	A >> P
AutoAlign	---
Phase oversampling	0 %
FoV read	192 mm
FoV phase	100.0 %
Slice thickness	1.50 mm
TR	1689 ms
TE	21.00 ms
Multi-band accel. factor	3
Filter	None
Coil elements	A32

Contrast - Common

TR	1689 ms
TE	21.00 ms
MTC	Off
Magn. preparation	None
Flip angle	55 deg
Fat suppr.	Fat sat.

Contrast - Dynamic

Averaging mode	Long term
Reconstruction	Magnitude
Measurements	5
Delay in TR	0 ms
Multiple series	Off

Resolution - Common

FoV read	192 mm
FoV phase	100.0 %
Slice thickness	1.50 mm
Base resolution	128
Phase resolution	100 %
Phase partial Fourier	7/8
Interpolation	Off

Resolution - iPAT

PAT mode	GRAPPA
Accel. factor PE	2
Ref. lines PE	32

Resolution - iPAT

Reference scan mode	GRE

Resolution - Filter Image

Distortion Corr.	Off
Prescan Normalize	Off

Resolution - Filter Rawdata

Raw filter	Off
Elliptical filter	Off
Hamming	Off

Geometry - Common

Slice group	1
Slices	96
Dist. factor	0 %
Position	L3.2 A34.5 H8.8 mm
Orientation	Transversal
Phase enc. dir.	A >> P
FoV read	192 mm
FoV phase	100.0 %
Slice thickness	1.50 mm
TR	1689 ms
Multi-slice mode	Interleaved
Series	Interleaved
Multi-band accel. factor	3

Geometry - AutoAlign

Slice group	1
Position	L3.2 A34.5 H8.8 mm
Orientation	Transversal
Phase enc. dir.	A >> P
AutoAlign	---
Initial Position	L3.2 A34.5 H8.8
Phase	-34.5 mm
Read	-3.2 mm
Shift	8.8 mm
Initial Rotation	0.00 deg
Initial Orientation	Transversal

Geometry - Saturation

Fat suppr.	Fat sat.
Special sat.	None

Geometry - Tim Planning Suite

Set-n-Go Protocol	Off
Table position	H
Table position	0 mm
Inline Composing	Off

System - Miscellaneous

Positioning mode	FIX
Table position	H
Table position	0 mm
MSMA	S - C - T
Sagittal	R >> L
Coronal	A >> P
Transversal	F >> H
Coil Combine Mode	Sum of Squares

System - Miscellaneous

Matrix Optimization	Off
AutoAlign	---
Coil Select Mode	Default

System - Adjustments

B0 Shim mode	Brain
B1 Shim mode	TrueForm
Confirm freq. adjustment	Off
Assume Dominant Fat	Off
Assume Silicone	Off
Adjustment Tolerance	Auto

System - Adjust Volume

! Position	L2.4 A33.6 H4.0 mm
! Orientation	Transversal
! Rotation	0.00 deg
! A >> P	211 mm
! R >> L	160 mm
! F >> H	149 mm
Reset	Off

System - Tx/Rx

Frequency 1H	297.230405 MHz
Correction factor	1
Gain	High
Img. Scale Cor.	0.500
Reset	Off
? Ref. amplitude 1H	0.000 V

Physio - Signal1

1st Signal/Mode	None
TR	1689 ms
Multi-band accel. factor	3

BOLD

GLM Statistics	Off
Dynamic t-maps	Off
Ignore meas. at start	0
Ignore after transition	0
Model transition states	On
Temp. highpass filter	On
Threshold	4.00
Paradigm size	20
Meas[1]	Baseline
Meas[2]	Baseline
Meas[3]	Baseline
Meas[4]	Baseline
Meas[5]	Baseline
Meas[6]	Baseline
Meas[7]	Baseline
Meas[8]	Baseline
Meas[9]	Baseline
Meas[10]	Baseline
Meas[11]	Active
Meas[12]	Active
Meas[13]	Active
Meas[14]	Active
Meas[15]	Active
Meas[16]	Active
Meas[17]	Active
Meas[18]	Active
Meas[19]	Active
Meas[20]	Active
Motion correction	Off

BOLD

Spatial filter	Off
Measurements	5
Delay in TR	0 ms
Multiple series	Off

Sequence - Part 1

Introduction	Off
Contrasts	1
Flow comp.	No
Multi-slice mode	Interleaved
Free echo spacing	Off
Echo spacing	0.62 ms
Bandwidth	1860 Hz/Px

Sequence - Part 2

EPI factor	128
Gradient mode	Fast
RF spoiling	Off

Sequence - Special

Excite pulse duration	3200 us
Single-band images	On
MB LeakBlock kernel	Off
MB dual kernel	Off
MB RF phase scramble	On
SENSE1 coil combine	On
Invert RO/PE polarity	On
PF omits higher k-space	Off
Disable freq. update	Off
Force equal slice timing	Off
Online multi-band recon.	Online
FFT scale factor	1.00
Fat saturation FA	110.0 deg
GRE iPAT ref. FA	12.0 deg
Physio recording	DICOM
Triggering scheme	Standard

cmrr_mbep2d_bold_3x2_1.5iso_100vols

TA: 3:16 PM: FIX Voxel size: 1.5×1.5×1.5 mmPAT: 2 Rel. SNR: 1.00 : epfid

Properties

Prio recon	Off
Load images to viewer	On
Inline movie	Off
Auto store images	On
Load images to stamp segments	Off
Load images to graphic segments	Off
Auto open inline display	Off
Auto close inline display	Off
Start measurement without further preparation	Off
Wait for user to start	Off
Start measurements	Single measurement

Routine

Slice group	1
Slices	96
Dist. factor	0 %
Position	L3.2 A34.5 H8.8 mm
Orientation	Transversal
Phase enc. dir.	A >> P
AutoAlign	---
Phase oversampling	0 %
FoV read	192 mm
FoV phase	100.0 %
Slice thickness	1.50 mm
TR	1689 ms
TE	21.00 ms
Multi-band accel. factor	3
Filter	None
Coil elements	A32

Contrast - Common

TR	1689 ms
TE	21.00 ms
MTC	Off
Magn. preparation	None
Flip angle	55 deg
Fat suppr.	Fat sat.

Contrast - Dynamic

Averaging mode	Long term
Reconstruction	Magnitude
Measurements	100
Delay in TR	0 ms
Multiple series	Off

Resolution - Common

FoV read	192 mm
FoV phase	100.0 %
Slice thickness	1.50 mm
Base resolution	128
Phase resolution	100 %
Phase partial Fourier	7/8
Interpolation	Off

Resolution - iPAT

PAT mode	GRAPPA
Accel. factor PE	2
Ref. lines PE	32

Resolution - iPAT

Reference scan mode	GRE

Resolution - Filter Image

Distortion Corr.	Off
Prescan Normalize	Off

Resolution - Filter Rawdata

Raw filter	Off
Elliptical filter	Off
Hamming	Off

Geometry - Common

Slice group	1
Slices	96
Dist. factor	0 %
Position	L3.2 A34.5 H8.8 mm
Orientation	Transversal
Phase enc. dir.	A >> P
FoV read	192 mm
FoV phase	100.0 %
Slice thickness	1.50 mm
TR	1689 ms
Multi-slice mode	Interleaved
Series	Interleaved
Multi-band accel. factor	3

Geometry - AutoAlign

Slice group	1
Position	L3.2 A34.5 H8.8 mm
Orientation	Transversal
Phase enc. dir.	A >> P
AutoAlign	---
Initial Position	L3.2 A34.5 H8.8
Phase	-34.5 mm
Read	-3.2 mm
Shift	8.8 mm
Initial Rotation	0.00 deg
Initial Orientation	Transversal

Geometry - Saturation

Fat suppr.	Fat sat.
Special sat.	None

Geometry - Tim Planning Suite

Set-n-Go Protocol	Off
Table position	H
Table position	0 mm
Inline Composing	Off

System - Miscellaneous

Positioning mode	FIX
Table position	H
Table position	0 mm
MSMA	S - C - T
Sagittal	R >> L
Coronal	A >> P
Transversal	F >> H
Coil Combine Mode	Sum of Squares

System - Miscellaneous

Matrix Optimization	Off
AutoAlign	---
Coil Select Mode	Default

System - Adjustments

B0 Shim mode	Brain
B1 Shim mode	TrueForm
Confirm freq. adjustment	Off
Assume Dominant Fat	Off
Assume Silicone	Off
Adjustment Tolerance	Auto

System - Adjust Volume

! Position	L2.4 A33.6 H4.0 mm
! Orientation	Transversal
! Rotation	0.00 deg
! A >> P	211 mm
! R >> L	160 mm
! F >> H	149 mm
Reset	Off

System - Tx/Rx

Frequency 1H	297.230405 MHz
Correction factor	1
Gain	High
Img. Scale Cor.	0.500
Reset	Off
? Ref. amplitude 1H	0.000 V

Physio - Signal1

1st Signal/Mode	None
TR	1689 ms
Multi-band accel. factor	3

BOLD

GLM Statistics	Off
Dynamic t-maps	Off
Ignore meas. at start	0
Ignore after transition	0
Model transition states	On
Temp. highpass filter	On
Threshold	4.00
Paradigm size	20
Meas[1]	Baseline
Meas[2]	Baseline
Meas[3]	Baseline
Meas[4]	Baseline
Meas[5]	Baseline
Meas[6]	Baseline
Meas[7]	Baseline
Meas[8]	Baseline
Meas[9]	Baseline
Meas[10]	Baseline
Meas[11]	Active
Meas[12]	Active
Meas[13]	Active
Meas[14]	Active
Meas[15]	Active
Meas[16]	Active
Meas[17]	Active
Meas[18]	Active
Meas[19]	Active
Meas[20]	Active
Motion correction	Off

BOLD

Spatial filter	Off
Measurements	100
Delay in TR	0 ms
Multiple series	Off

Sequence - Part 1

Introduction	Off
Contrasts	1
Flow comp.	No
Multi-slice mode	Interleaved
Free echo spacing	Off
Echo spacing	0.62 ms
Bandwidth	1860 Hz/Px

Sequence - Part 2

EPI factor	128
Gradient mode	Fast
RF spoiling	Off

Sequence - Special

Excite pulse duration	3200 us
Single-band images	On
MB LeakBlock kernel	Off
MB dual kernel	Off
MB RF phase scramble	On
SENSE1 coil combine	On
Invert RO/PE polarity	Off
PF omits higher k-space	Off
Disable freq. update	Off
Force equal slice timing	Off
Online multi-band recon.	Online
FFT scale factor	1.00
Fat saturation FA	110.0 deg
GRE iPAT ref. FA	12.0 deg
Physio recording	DICOM
Triggering scheme	Standard

fastestmap_linear_3
TA: 0:13 PM: REF VoI: 12 ×20 ×12 mmRel. SNR: 1.00 : fastmp

Properties

Prio recon	Off
Load images to viewer	On
Inline movie	Off
Auto store images	On
Load images to stamp segments	Off
Load images to graphic segments	Off
Auto open inline display	On
Auto close inline display	Off
Start measurement without further preparation	Off
Wait for user to start	Off
Start measurements	Single measurement

Routine

Position	R0.3 A22.3 F44.3 mm
Orientation	C > T-5.3
Rotation	0 deg
VoI F >> H	20 mm
VoI F >> H	20 mm
VoI A >> P	12 mm
TR	4169 ms
TE	48.70 ms
Averages	1
Filter	None
Coil elements	A32

Contrast

TR	4169 ms
TE	48.70 ms
Tau	5.00 ms
Averages	1
Excite flip angle	90 deg
Refocus flip angle	180 deg
Measurements	1

Resolution - Common

Vector size	256

Geometry - Common

Position	R0.3 A22.3 F44.3 mm
Orientation	C > T-5.3
Rotation	0 deg
VoI F >> H	20 mm
VoI R >> L	12 mm
VoI A >> P	12 mm

Geometry - AutoAlign

AutoAlign	---
Initial Position	R0.3 A22.3 F44.3
Phase	-0.3 mm
Read	42.1 mm
Shift	-26.4 mm
Initial Rotation	0.00 deg
Initial Orientation	C > T
C > T	-5.3
> S	0.0

System - Miscellaneous

Positioning mode	REF
Table position	H
Table position	0 mm
MSMA	S - C - T
Sagittal	R >> L
Coronal	A >> P
Transversal	F >> H
Save uncombined	Off
AutoAlign	---
Coil Select Mode	Default

System - Adjustments

B0 Shim mode	Tune up
B1 Shim mode	TrueForm
Adj. water suppr.	Off
Confirm freq. adjustment	Off
Assume Dominant Fat	Off
Assume Silicone	Off
Adjustment Tolerance	Auto

System - Adjust Volume

Position	R0.3 A22.3 F44.3 mm
Orientation	C > T-5.3
Rotation	0.00 deg
R >> L	12 mm
F >> H	20 mm
A >> P	12 mm
Reset	Off

System - Tx/Rx

Frequency 1H	297.230405 MHz
Correction factor	1
Gain	High
Img. Scale Cor.	1.000
Reset	Off
? Ref. amplitude 1H	0.000 V

Physio - Signal1

1st Signal/Mode	None
TR	4169 ms

Sequence - Common

Delta frequency	0.0 ppm
Phase cycling	None
Bandwidth	100000 Hz
Acquisition duration	2 ms

Sequence - Special

Type of fit	Linear 3-proj
VoI fit factor	100 %
Force spherical fit VoI	Off Force spherical fit VoI
Save plots to database	On
Refocus pulses	Normal
Excite pulse duration	6400 us
Refocus pulse duration	6400 us
Bar FoV	384 mm
Bar thickness	2.5 mm
Inversion pulse	Off
Multi-echo acquisition	On
Number of echoes	8

**fastestmap_full_6**
TA: 0:25 PM: FIX Vol: 12 ×20 ×12 mmRel. SNR: 1.00 : fastmp

Properties

Prio recon	Off
Load images to viewer	On
Inline movie	Off
Auto store images	On
Load images to stamp segments	Off
Load images to graphic segments	Off
Auto open inline display	On
Auto close inline display	Off
Start measurement without further preparation	Off
Wait for user to start	Off
Start measurements	Single measurement

Routine

Position	R0.3 A22.3 F44.3 mm
Orientation	C > T-5.3
Rotation	0 deg
VoI F >> H	20 mm
VoI F >> H	20 mm
VoI A >> P	12 mm
TR	4169 ms
TE	48.70 ms
Averages	1
Filter	None
Coil elements	A32

Contrast

TR	4169 ms
TE	48.70 ms
Tau	5.00 ms
Averages	1
Excite flip angle	90 deg
Refocus flip angle	180 deg
Measurements	1

Resolution - Common

Vector size	256

Geometry - Common

Position	R0.3 A22.3 F44.3 mm
Orientation	C > T-5.3
Rotation	0 deg
VoI F >> H	20 mm
VoI R >> L	12 mm
VoI A >> P	12 mm

Geometry - AutoAlign

AutoAlign	---
Initial Position	R0.3 A22.3 F44.3
Phase	-0.3 mm
Read	42.1 mm
Shift	-26.4 mm
Initial Rotation	0.00 deg
Initial Orientation	C > T
C > T	-5.3
> S	0.0

System - Miscellaneous

Positioning mode	FIX
Table position	H

System - Miscellaneous

Table position	0 mm
MSMA	S - C - T
Sagittal	R >> L
Coronal	A >> P
Transversal	F >> H
Save uncombined	Off
AutoAlign	---
Coil Select Mode	Default

System - Adjustments

B0 Shim mode	Tune up
B1 Shim mode	TrueForm
Adj. water suppr.	Off
Confirm freq. adjustment	Off
Assume Dominant Fat	Off
Assume Silicone	Off
Adjustment Tolerance	Auto

System - Adjust Volume

! Position	R0.3 A22.3 F44.3 mm
! Orientation	C > T-5.3
! Rotation	0.00 deg
! R >> L	12 mm
! F >> H	20 mm
! A >> P	12 mm
Reset	Off

System - Tx/Rx

Frequency 1H	297.230405 MHz
Correction factor	1
Gain	High
Img. Scale Cor.	1.000
Reset	Off
? Ref. amplitude 1H	0.000 V

Physio - Signal1

1st Signal/Mode	None
TR	4169 ms

Sequence - Common

Delta frequency	0.0 ppm
Phase cycling	None
Bandwidth	100000 Hz
Acquisition duration	2 ms

Sequence - Special

Type of fit	Full 6-proj
VoI fit factor	100 %
Force spherical fit VoI	Off Force spherical fit VoI
Save plots to database	On
Refocus pulses	Normal
Excite pulse duration	6400 us
Refocus pulse duration	6400 us
Bar FoV	384 mm
Bar thickness	2.5 mm
Inversion pulse	Off
Multi-echo acquisition	On
Number of echoes	8

fastestmap_full_6
TA: 0:25 PM: FIX Vol: 12 ×20 ×12 mmRel. SNR: 1.00 : fastmp

Properties

Prio recon	Off
Load images to viewer	On
Inline movie	Off
Auto store images	On
Load images to stamp segments	Off
Load images to graphic segments	Off
Auto open inline display	On
Auto close inline display	Off
Start measurement without further preparation	Off
Wait for user to start	Off
Start measurements	Single measurement

Routine

Position	R0.3 A22.3 F44.3 mm
Orientation	C > T-5.3
Rotation	0 deg
VoI F >> H	20 mm
VoI F >> H	20 mm
VoI A >> P	12 mm
TR	4169 ms
TE	48.70 ms
Averages	1
Filter	None
Coil elements	A32

Contrast

TR	4169 ms
TE	48.70 ms
Tau	5.00 ms
Averages	1
Excite flip angle	90 deg
Refocus flip angle	180 deg
Measurements	1

Resolution - Common

Vector size	256

Geometry - Common

Position	R0.3 A22.3 F44.3 mm
Orientation	C > T-5.3
Rotation	0 deg
VoI F >> H	20 mm
VoI R >> L	12 mm
VoI A >> P	12 mm

Geometry - AutoAlign

AutoAlign	---
Initial Position	R0.3 A22.3 F44.3
Phase	-0.3 mm
Read	42.1 mm
Shift	-26.4 mm
Initial Rotation	0.00 deg
Initial Orientation	C > T
C > T	-5.3
> S	0.0

System - Miscellaneous

Positioning mode	FIX
Table position	H

System - Miscellaneous

Table position	0 mm
MSMA	S - C - T
Sagittal	R >> L
Coronal	A >> P
Transversal	F >> H
Save uncombined	Off
AutoAlign	---
Coil Select Mode	Default

System - Adjustments

B0 Shim mode	Tune up
B1 Shim mode	TrueForm
Adj. water suppr.	Off
Confirm freq. adjustment	Off
Assume Dominant Fat	Off
Assume Silicone	Off
Adjustment Tolerance	Auto

System - Adjust Volume

! Position	R0.3 A22.3 F44.3 mm
! Orientation	C > T-5.3
! Rotation	0.00 deg
! R >> L	12 mm
! F >> H	20 mm
! A >> P	12 mm
Reset	Off

System - Tx/Rx

Frequency 1H	297.230405 MHz
Correction factor	1
Gain	High
Img. Scale Cor.	1.000
Reset	Off
? Ref. amplitude 1H	0.000 V

Physio - Signal1

1st Signal/Mode	None
TR	4169 ms

Sequence - Common

Delta frequency	0.0 ppm
Phase cycling	None
Bandwidth	100000 Hz
Acquisition duration	2 ms

Sequence - Special

Type of fit	Full 6-proj
VoI fit factor	100 %
Force spherical fit VoI	Off Force spherical fit VoI
Save plots to database	On
Refocus pulses	Normal
Excite pulse duration	6400 us
Refocus pulse duration	6400 us
Bar FoV	384 mm
Bar thickness	2.5 mm
Inversion pulse	Off
Multi-echo acquisition	On
Number of echoes	8

fastestmap_full_6
TA: 0:25 PM: FIX VoI: 12 ×20 ×12 mmRel. SNR: 1.00 : fastmp

Properties

Prio recon	Off
Load images to viewer	On
Inline movie	Off
Auto store images	On
Load images to stamp segments	Off
Load images to graphic segments	Off
Auto open inline display	On
Auto close inline display	Off
Start measurement without further preparation	Off
Wait for user to start	Off
Start measurements	Single measurement

Routine

Position	R0.3 A22.3 F44.3 mm
Orientation	C > T-5.3
Rotation	0 deg
VoI F >> H	20 mm
VoI F >> H	20 mm
VoI A >> P	12 mm
TR	4169 ms
TE	48.70 ms
Averages	1
Filter	None
Coil elements	A32

Contrast

TR	4169 ms
TE	48.70 ms
Tau	5.00 ms
Averages	1
Excite flip angle	90 deg
Refocus flip angle	180 deg
Measurements	1

Resolution - Common

Vector size	256

Geometry - Common

Position	R0.3 A22.3 F44.3 mm
Orientation	C > T-5.3
Rotation	0 deg
VoI F >> H	20 mm
VoI R >> L	12 mm
VoI A >> P	12 mm

Geometry - AutoAlign

AutoAlign	---
Initial Position	R0.3 A22.3 F44.3
Phase	-0.3 mm
Read	42.1 mm
Shift	-26.4 mm
Initial Rotation	0.00 deg
Initial Orientation	C > T
C > T	-5.3
> S	0.0

System - Miscellaneous

Positioning mode	FIX
Table position	H

System - Miscellaneous

Table position	0 mm
MSMA	S - C - T
Sagittal	R >> L
Coronal	A >> P
Transversal	F >> H
Save uncombined	Off
AutoAlign	---
Coil Select Mode	Default

System - Adjustments

B0 Shim mode	Tune up
B1 Shim mode	TrueForm
Adj. water suppr.	Off
Confirm freq. adjustment	Off
Assume Dominant Fat	Off
Assume Silicone	Off
Adjustment Tolerance	Auto

System - Adjust Volume

! Position	R0.3 A22.3 F44.3 mm
! Orientation	C > T-5.3
! Rotation	0.00 deg
! R >> L	12 mm
! F >> H	20 mm
! A >> P	12 mm
Reset	Off

System - Tx/Rx

Frequency 1H	297.230405 MHz
Correction factor	1
Gain	High
Img. Scale Cor.	1.000
Reset	Off
? Ref. amplitude 1H	0.000 V

Physio - Signal1

1st Signal/Mode	None
TR	4169 ms

Sequence - Common

Delta frequency	0.0 ppm
Phase cycling	None
Bandwidth	100000 Hz
Acquisition duration	2 ms

Sequence - Special

Type of fit	Full 6-proj
VoI fit factor	100 %
Force spherical fit VoI	Off Force spherical fit VoI
Save plots to database	On
Refocus pulses	Normal
Excite pulse duration	6400 us
Refocus pulse duration	6400 us
Bar FoV	384 mm
Bar thickness	2.5 mm
Inversion pulse	Off
Multi-echo acquisition	On
Number of echoes	8

**fastestmap_linear_6**
TA: 0:50 PM: FIX VoI: 12 ×20 ×12 mmRel. SNR: 1.00 : fastmp

Properties

Prio recon	Off
Load images to viewer	On
Inline movie	Off
Auto store images	On
Load images to stamp segments	Off
Load images to graphic segments	Off
Auto open inline display	On
Auto close inline display	Off
Start measurement without further preparation	Off
Wait for user to start	Off
Start measurements	Single measurement

Routine

Position	R0.3 A22.3 F44.3 mm
Orientation	C > T-5.3
Rotation	0 deg
VoI F >> H	20 mm
VoI F >> H	20 mm
VoI A >> P	12 mm
TR	4169 ms
TE	48.70 ms
Averages	1
Filter	None
Coil elements	A32

Contrast

TR	4169 ms
TE	48.70 ms
Tau	10.00 ms
Averages	1
Excite flip angle	90 deg
Refocus flip angle	180 deg
Measurements	1

Resolution - Common

Vector size	256

Geometry - Common

Position	R0.3 A22.3 F44.3 mm
Orientation	C > T-5.3
Rotation	0 deg
VoI F >> H	20 mm
VoI R >> L	12 mm
VoI A >> P	12 mm

Geometry - AutoAlign

AutoAlign	---
Initial Position	R0.3 A22.3 F44.3
Phase	-0.3 mm
Read	42.1 mm
Shift	-26.4 mm
Initial Rotation	0.00 deg
Initial Orientation	C > T
C > T	-5.3
> S	0.0

System - Miscellaneous

Positioning mode	FIX
Table position	H

System - Miscellaneous

Table position	0 mm
MSMA	S - C - T
Sagittal	R >> L
Coronal	A >> P
Transversal	F >> H
Save uncombined	Off
AutoAlign	---
Coil Select Mode	Default

System - Adjustments

B0 Shim mode	Tune up
B1 Shim mode	TrueForm
Adj. water suppr.	Off
Confirm freq. adjustment	Off
Assume Dominant Fat	Off
Assume Silicone	Off
Adjustment Tolerance	Auto

System - Adjust Volume

! Position	R0.3 A22.3 F44.3 mm
! Orientation	C > T-5.3
! Rotation	0.00 deg
! R >> L	12 mm
! F >> H	20 mm
! A >> P	12 mm
Reset	Off

System - Tx/Rx

Frequency 1H	297.230405 MHz
Correction factor	1
Gain	High
Img. Scale Cor.	1.000
Reset	Off
? Ref. amplitude 1H	0.000 V

Physio - Signal1

1st Signal/Mode	None
TR	4169 ms

Sequence - Common

Delta frequency	0.0 ppm
Phase cycling	None
Bandwidth	100000 Hz
Acquisition duration	2 ms

Sequence - Special

Type of fit	Linear 6-proj
VoI fit factor	100 %
Force spherical fit VoI	Off Force spherical fit VoI
Save plots to database	On
Refocus pulses	Normal
Excite pulse duration	6400 us
Refocus pulse duration	6400 us
Bar FoV	300 mm
Bar thickness	5.0 mm
Inversion pulse	Off
Multi-echo acquisition	Off

sLaser_LW
TA: 0:15 PM: FIX Vol: 12 ×20 ×12 mmRel. SNR: 1.00 : sead

Properties

Prio recon	Off
Load images to viewer	On
Inline movie	Off
Auto store images	On
Load images to stamp segments	Off
Load images to graphic segments	Off
Auto open inline display	On
Auto close inline display	Off
Start measurement without further preparation	Off
Wait for user to start	Off
Start measurements	Single measurement

Routine

Position	R0.3 A22.3 F44.3 mm
Orientation	C > T-5.3
Rotation	0 deg
Vol F >> H	20 mm
Vol F >> H	20 mm
Vol A >> P	12 mm
TR	5000 ms
TE 1	7 ms
TE 2	10 ms
TE 3	9 ms
Averages	1
Filter	None
Coil elements	A32

Contrast

TR	5000 ms
TE 1	7 ms
TE 2	10 ms
TE 3	9 ms
Total TE	26 ms
Averages	1
Excitation Flip angle	90 deg
Refocusing Flip angle	180 deg
VAPOR WS	None

Resolution - Common

Prescan Normalize	Off
Vector size	2048

Geometry - Common

Position	R0.3 A22.3 F44.3 mm
Orientation	C > T-5.3
Rotation	0 deg
Vol F >> H	20 mm
Vol R >> L	12 mm
Vol A >> P	12 mm

Geometry - AutoAlign

AutoAlign	---
Initial Position	R0.3 A22.3 F44.3
Phase	-0.3 mm
Read	42.1 mm
Shift	-26.4 mm
Initial Rotation	0.00 deg
Initial Orientation	C > T
C > T	-5.3

Geometry - AutoAlign

> S	0.0

System - Miscellaneous

Positioning mode	FIX
Table position	H
Table position	0 mm
MSMA	S - C - T
Sagittal	R >> L
Coronal	A >> P
Transversal	F >> H
Save uncombined	Off
Save single averages	On
AutoAlign	---
Coil Select Mode	Default

System - Adjustments

B0 Shim mode	Tune up
B1 Shim mode	TrueForm
Confirm freq. adjustment	Off
Assume Dominant Fat	Off
Assume Silicone	Off
Adjustment Tolerance	Auto

System - Adjust Volume

! Position	R0.3 A22.3 F44.3 mm
! Orientation	C > T-5.3
! Rotation	0.00 deg
! R >> L	12 mm
! F >> H	20 mm
! A >> P	12 mm
Reset	Off

System - Tx/Rx

Frequency 1H	297.230405 MHz
Correction factor	1
Gain	High
Img. Scale Cor.	1.000
Reset	Off
? Ref. amplitude 1H	0.000 V

Physio - Signal1

1st Signal/Mode	None
TR	5000 ms

Sequence - Common

Preparation scans	2
Delta frequency	0.0 ppm
Phase cycling	Auto
Bandwidth	6000 Hz
Acquisition duration	341 ms
Remove oversampling	On

Sequence - Special

Excitation duration	1500 us
Refocusing duration	3500 us
AFP Type	GOIA-WURST
Bandwidth_1ms	45 kHz
HSn modulation	16
Gradient factor	85 %
GOIA/FOCI	On

Sequence - Special

Inversion pulse	Off
FA AutoCalib	Off
Advanced User	Off
Calibration Type	None
Debug Type	None
Gradient Max. Amplitude	42 mT/m
Ramp time	210 us

svs_slaser_dkd_FA_cal

TA: 1:12 PM: FIX Vol: 12 ×20 ×12 mmRel. SNR: 1.00 : sead

Properties

Prio recon	Off
Load images to viewer	On
Inline movie	Off
Auto store images	On
Load images to stamp segments	Off
Load images to graphic segments	Off
Auto open inline display	Off
Auto close inline display	Off
Start measurement without further preparation	Off
Wait for user to start	Off
Start measurements	Single measurement

Routine

Position	R0.3 A22.3 F44.3 mm
Orientation	C > T-5.3
Rotation	0 deg
Vol F >> H	20 mm
Vol F >> H	20 mm
Vol A >> P	12 mm
TR	8000 ms
TE 1	10 ms
TE 2	15 ms
TE 3	10 ms
Averages	1
Filter	None
Coil elements	A32

Contrast

TR	8000 ms
TE 1	10 ms
TE 2	15 ms
TE 3	10 ms
Total TE	35 ms
Averages	1
Excitation Flip angle	50 deg
Refocusing Flip angle	180 deg
VAPOR WS	None

Resolution - Common

Prescan Normalize	Off
Vector size	2048

Geometry - Common

Position	R0.3 A22.3 F44.3 mm
Orientation	C > T-5.3
Rotation	0 deg
Vol F >> H	20 mm
Vol R >> L	12 mm
Vol A >> P	12 mm

Geometry - AutoAlign

AutoAlign	---
Initial Position	R0.3 A22.3 F44.3
Phase	-0.3 mm
Read	42.1 mm
Shift	-26.4 mm
Initial Rotation	0.00 deg
Initial Orientation	C > T
C > T	-5.3

Geometry - AutoAlign

> S	0.0

System - Miscellaneous

Positioning mode	FIX
Table position	H
Table position	0 mm
MSMA	S - C - T
Sagittal	R >> L
Coronal	A >> P
Transversal	F >> H
Save uncombined	Off
Save single averages	On
AutoAlign	---
Coil Select Mode	Default

System - Adjustments

B0 Shim mode	Tune up
B1 Shim mode	TrueForm
Confirm freq. adjustment	Off
Assume Dominant Fat	Off
Assume Silicone	Off
Adjustment Tolerance	Auto

System - Adjust Volume

! Position	R0.3 A22.3 F44.3 mm
! Orientation	C > T-5.3
! Rotation	0.00 deg
! R >> L	12 mm
! F >> H	20 mm
! A >> P	12 mm
Reset	Off

System - Tx/Rx

Frequency 1H	297.230405 MHz
Correction factor	1
Gain	High
Img. Scale Cor.	1.000
Reset	Off
! Ref. amplitude 1H	225.000 V

Physio - Signal1

1st Signal/Mode	None
TR	8000 ms

Sequence - Common

Preparation scans	1
Delta frequency	-2.0 ppm
Phase cycling	Auto
Bandwidth	6000 Hz
Acquisition duration	341 ms
Remove oversampling	On

Sequence - Special

Excitation duration	1500 us
Refocusing duration	3500 us
AFP Type	GOIA-WURST
Bandwidth_1ms	45 kHz
HSn modulation	16
Gradient factor	85 %
GOIA/FOCI	On

Sequence - Special

Inversion pulse	Off
FA AutoCalib	Off
Advanced User	Off
Calibration Type	Excitation FA
Debug Type	None
Increment step size	10 deg
Measurements	8
Gradient Max. Amplitude	42 mT/m
Ramp time	210 us

sLaser_WSCalib_coarse
TA: 1:45 PM: FIX Vol: 12 ×20 ×12 mmRel. SNR: 1.00 : sead

Properties

Prio recon	Off
Load images to viewer	On
Inline movie	Off
Auto store images	On
Load images to stamp segments	Off
Load images to graphic segments	Off
Auto open inline display	On
Auto close inline display	Off
Start measurement without further preparation	Off
Wait for user to start	Off
Start measurements	Single measurement

Routine

Position	R0.3 A22.3 F44.3 mm
Orientation	C > T-5.3
Rotation	0 deg
Vol F >> H	20 mm
Vol F >> H	20 mm
Vol A >> P	12 mm
TR	5000 ms
TE 1	7 ms
TE 2	10 ms
TE 3	9 ms
Averages	1
Filter	None
Coil elements	A32

Contrast

TR	5000 ms
TE 1	7 ms
TE 2	10 ms
TE 3	9 ms
Total TE	26 ms
Averages	1
Excitation Flip angle	90 deg
Refocusing Flip angle	180 deg
VAPOR WS	Enabled
Water suppr. BW	135 Hz
VAPOR Flip angle	10 deg

Resolution - Common

Prescan Normalize	Off
Vector size	2048

Geometry - Common

Position	R0.3 A22.3 F44.3 mm
Orientation	C > T-5.3
Rotation	0 deg
Vol F >> H	20 mm
Vol R >> L	12 mm
Vol A >> P	12 mm

Geometry - AutoAlign

AutoAlign	---
Initial Position	R0.3 A22.3 F44.3
Phase	-0.3 mm
Read	42.1 mm
Shift	-26.4 mm
Initial Rotation	0.00 deg

Geometry - AutoAlign

Initial Orientation	C > T
C > T	-5.3
> S	0.0

System - Miscellaneous

Positioning mode	FIX
Table position	H
Table position	0 mm
MSMA	S - C - T
Sagittal	R >> L
Coronal	A >> P
Transversal	F >> H
Save uncombined	Off
Save single averages	On
AutoAlign	---
Coil Select Mode	Default

System - Adjustments

B0 Shim mode	Tune up
B1 Shim mode	TrueForm
Confirm freq. adjustment	Off
Assume Dominant Fat	Off
Assume Silicone	Off
Adjustment Tolerance	Auto

System - Adjust Volume

! Position	R0.3 A22.3 F44.3 mm
! Orientation	C > T-5.3
! Rotation	0.00 deg
! R >> L	12 mm
! F >> H	20 mm
! A >> P	12 mm
Reset	Off

System - Tx/Rx

Frequency 1H	297.230405 MHz
Correction factor	1
Gain	High
Img. Scale Cor.	1.000
Reset	Off
? Ref. amplitude 1H	0.000 V

Physio - Signal1

1st Signal/Mode	None
TR	5000 ms

Sequence - Common

Preparation scans	1
Delta frequency	-2.0 ppm
Water Ref. Scan	Off
Phase cycling	Auto
Bandwidth	6000 Hz
Acquisition duration	341 ms
Remove oversampling	On

Sequence - Special

Excitation duration	1500 us
Refocusing duration	3500 us
AFP Type	GOIA-WURST
Bandwidth_1ms	45 kHz

Sequence - Special

HSn modulation	16
Gradient factor	85 %
GOIA/FOCI	On
Inversion pulse	Off
FA AutoCalib	Off
Advanced User	Off
OVS module 1	On
OVS module 2	On
OVS module 3	On
OVS module 4	On
OVS slab X	120 mm
OVS slab Y up	120 mm
OVS slab Y down	120 mm
OVS slab Z	120 mm
OVS gap	7 mm
OVS delay 7	88 ms
OVS delay 8	21 ms
Calibration Type	VAPOR WS FA
Debug Type	None
Increment step size	10 deg
Measurements	20
Gradient Max. Amplitude	42 mT/m
Ramp time	210 us

**sLaser_WSCalib_fine**
TA: 0:45 PM: FIX Vol: 12 ×20 ×12 mmRel. SNR: 1.00 : sead

Properties

Prio recon	Off
Load images to viewer	On
Inline movie	Off
Auto store images	On
Load images to stamp segments	Off
Load images to graphic segments	Off
Auto open inline display	On
Auto close inline display	Off
Start measurement without further preparation	Off
Wait for user to start	Off
Start measurements	Single measurement

Routine

Position	R0.3 A22.3 F44.3 mm
Orientation	C > T-5.3
Rotation	0 deg
Vol F >> H	20 mm
Vol F >> H	20 mm
Vol A >> P	12 mm
TR	5000 ms
TE 1	7 ms
TE 2	10 ms
TE 3	9 ms
Averages	1
Filter	None
Coil elements	A32

Contrast

TR	5000 ms
TE 1	7 ms
TE 2	10 ms
TE 3	9 ms
Total TE	26 ms
Averages	1
Excitation Flip angle	90 deg
Refocusing Flip angle	180 deg
VAPOR WS	Enabled
Water suppr. BW	135 Hz
VAPOR Flip angle	65 deg

Resolution - Common

Prescan Normalize	Off
Vector size	2048

Geometry - Common

Position	R0.3 A22.3 F44.3 mm
Orientation	C > T-5.3
Rotation	0 deg
Vol F >> H	20 mm
Vol R >> L	12 mm
Vol A >> P	12 mm

Geometry - AutoAlign

AutoAlign	---
Initial Position	R0.3 A22.3 F44.3
Phase	-0.3 mm
Read	42.1 mm
Shift	-26.4 mm
Initial Rotation	0.00 deg

Geometry - AutoAlign

Initial Orientation	C > T
C > T	-5.3
> S	0.0

System - Miscellaneous

Positioning mode	FIX
Table position	H
Table position	0 mm
MSMA	S - C - T
Sagittal	R >> L
Coronal	A >> P
Transversal	F >> H
Save uncombined	Off
Save single averages	On
AutoAlign	---
Coil Select Mode	Default

System - Adjustments

B0 Shim mode	Tune up
B1 Shim mode	TrueForm
Confirm freq. adjustment	Off
Assume Dominant Fat	Off
Assume Silicone	Off
Adjustment Tolerance	Auto

System - Adjust Volume

! Position	R0.3 A22.3 F44.3 mm
! Orientation	C > T-5.3
! Rotation	0.00 deg
! R >> L	12 mm
! F >> H	20 mm
! A >> P	12 mm
Reset	Off

System - Tx/Rx

Frequency 1H	297.230405 MHz
Correction factor	1
Gain	High
Img. Scale Cor.	1.000
Reset	Off
? Ref. amplitude 1H	0.000 V

Physio - Signal1

1st Signal/Mode	None
TR	5000 ms

Sequence - Common

Preparation scans	1
Delta frequency	-2.0 ppm
Water Ref. Scan	Off
Phase cycling	Auto
Bandwidth	6000 Hz
Acquisition duration	341 ms
Remove oversampling	On

Sequence - Special

Excitation duration	1500 us
Refocusing duration	3500 us
AFP Type	GOIA-WURST
Bandwidth_1ms	45 kHz

Sequence - Special

HSn modulation	16
Gradient factor	85 %
GOIA/FOCI	On
Inversion pulse	Off
FA AutoCalib	Off
Advanced User	Off
OVS module 1	On
OVS module 2	On
OVS module 3	On
OVS module 4	On
OVS slab X	120 mm
OVS slab Y up	120 mm
OVS slab Y down	120 mm
OVS slab Z	120 mm
OVS gap	7 mm
OVS delay 7	88 ms
OVS delay 8	21 ms
Calibration Type	VAPOR WS FA
Debug Type	None
Increment step size	2 deg
Measurements	8
Gradient Max. Amplitude	42 mT/m
Ramp time	210 us

sLaser_WS120_Brainstem

TA: 10:45 PM: FIX Vol: 12 ×20 ×12 mmRel. SNR: 1.00 : sead

Properties

Prio recon	Off
Load images to viewer	On
Inline movie	Off
Auto store images	On
Load images to stamp segments	Off
Load images to graphic segments	Off
Auto open inline display	On
Auto close inline display	Off
Start measurement without further preparation	Off
Wait for user to start	Off
Start measurements	Single measurement

Routine

Position	R0.3 A22.3 F44.3 mm
Orientation	C > T-5.3
Rotation	0 deg
Vol F >> H	20 mm
Vol F >> H	20 mm
Vol A >> P	12 mm
TR	5000 ms
TE 1	7 ms
TE 2	10 ms
TE 3	9 ms
Averages	120
Filter	None
Coil elements	A32

Contrast

TR	5000 ms
TE 1	7 ms
TE 2	10 ms
TE 3	9 ms
Total TE	26 ms
Averages	120
Excitation Flip angle	90 deg
Refocusing Flip angle	180 deg
VAPOR WS	Enabled
Water suppr. BW	135 Hz
VAPOR Flip angle	65 deg

Resolution - Common

Prescan Normalize	Off
Vector size	2048

Geometry - Common

Position	R0.3 A22.3 F44.3 mm
Orientation	C > T-5.3
Rotation	0 deg
Vol F >> H	20 mm
Vol R >> L	12 mm
Vol A >> P	12 mm

Geometry - AutoAlign

AutoAlign	---
Initial Position	R0.3 A22.3 F44.3
Phase	-0.3 mm
Read	42.1 mm
Shift	-26.4 mm
Initial Rotation	0.00 deg

Geometry - AutoAlign

Initial Orientation	C > T
C > T	-5.3
> S	0.0

System - Miscellaneous

Positioning mode	FIX
Table position	H
Table position	0 mm
MSMA	S - C - T
Sagittal	R >> L
Coronal	A >> P
Transversal	F >> H
Save uncombined	Off
Save single averages	On
AutoAlign	---
Coil Select Mode	Default

System - Adjustments

B0 Shim mode	Tune up
B1 Shim mode	TrueForm
Confirm freq. adjustment	Off
Assume Dominant Fat	Off
Assume Silicone	Off
Adjustment Tolerance	Auto

System - Adjust Volume

! Position	R0.3 A22.3 F44.3 mm
! Orientation	C > T-5.3
! Rotation	0.00 deg
! R >> L	12 mm
! F >> H	20 mm
! A >> P	12 mm
Reset	Off

System - Tx/Rx

Frequency 1H	297.230405 MHz
Correction factor	1
Gain	High
Img. Scale Cor.	1.000
Reset	Off
? Ref. amplitude 1H	0.000 V

Physio - Signal1

1st Signal/Mode	None
TR	5000 ms

Sequence - Common

Preparation scans	1
Delta frequency	-2.0 ppm
Water Ref. Scan	Enabled
No. of ref. scans	2
Phase cycling	Auto
Bandwidth	6000 Hz
Acquisition duration	341 ms
Remove oversampling	On

Sequence - Special

Excitation duration	1500 us
Refocusing duration	3500 us
AFP Type	GOIA-WURST

Sequence - Special

Bandwidth_1ms	45 kHz
HSn modulation	16
Gradient factor	85 %
GOIA/FOCI	On
Inversion pulse	Off
FA AutoCalib	Off
Advanced User	Off
OVS module 1	On
OVS module 2	On
OVS module 3	On
OVS module 4	On
OVS slab X	120 mm
OVS slab Y up	120 mm
OVS slab Y down	120 mm
OVS slab Z	120 mm
OVS gap	7 mm
OVS delay 7	88 ms
OVS delay 8	21 ms
Calibration Type	None
Debug Type	None
Gradient Max. Amplitude	42 mT/m
Ramp time	210 us
